# A systems biology approach identifies the role of dysregulated PRDM6 in the development of hypertension

**DOI:** 10.1172/JCI160036

**Published:** 2023-02-15

**Authors:** Kushan L. Gunawardhana, Lingjuan Hong, Trojan Rugira, Severin Uebbing, Joanna Kucharczak, Sameet Mehta, Dineth R. Karunamuni, Brenda Cabera-Mendoza, Neeru Gandotra, Curt Scharfe, Renato Polimanti, James P. Noonan, Arya Mani

**Affiliations:** 1 Cardiovascular Research Center, Department of Internal Medicine, and; 2 Department of Genetics, Yale University School of Medicine, New Haven, Connecticut, USA.; 3 Trinity Hall College, University of Cambridge, Cambridge, United Kingdom.; 4 Department of Psychiatry, Yale University School of Medicine, New Haven, Connecticut, USA.

**Keywords:** Cardiology, Nephrology, Genetic diseases, Hypertension, Neuroendocrine regulation

## Abstract

Genetic variants in the third intron of the *PRDM6* gene have been associated with BP traits in multiple GWAS. By combining fine mapping, massively parallel reporter assays, and gene editing, we identified super enhancers that drive the expression of *PRDM6* and are partly regulated by STAT1 as the causal variants for hypertension. The heterozygous disruption of *Prdm6* in mice expressing Cre recombinase under the control of mouse smooth muscle cell protein 22-α promoter (*Prdm6^fl/+^ SM22-Cre*) exhibited a markedly higher number of renin-producing cells in the kidneys at E18.5 compared with WT littermates and developed salt-induced systemic hypertension that was completely responsive to the renin inhibitor aliskiren. Strikingly, RNA-Seq analysis of the mouse aortas identified a network of PRDM6-regulated genes that are located in GWAS-associated loci for blood pressure, most notably *Sox6*, which modulates renin expression in the kidney. Accordingly, the smooth muscle cell–specific disruption of Sox6 in *Prdm6^fl/+^ SM22-Cre* mice resulted in a dramatic reduction of renin. Fate mapping and histological studies also showed increased numbers of neural crest–derived cells accompanied by increased collagen deposition in the kidneys of *Prdm6^fl/+^ Wnt1Cre-ZsGreen1Cre* mice compared with WT mice. These findings establish the role of PRDM6 as a regulator of renin-producing cell differentiation into smooth muscle cells and as an attractive target for the development of antihypertensive drugs.

## Graphical abstract

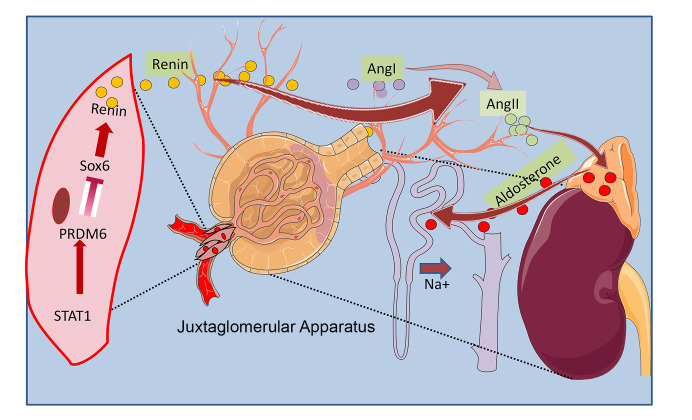

## Introduction

Systemic vascular hypertension is the largest modifiable risk factor for cardiovascular diseases worldwide (1). Despite intensive investment in developing antihypertensive drugs, about 50% of individuals with hypertension fail to reach the target BP (2), indicating an urgent need for the discovery of novel therapeutic targets.

BP has a significant heritable component accounting for 30%–50% of its variation (3). The identification of genetic variants influencing BP has great potential for the discovery of novel and potent therapeutic targets (4). Genome-wide discovery analyses of BP traits — systolic BP (SBP), diastolic BP (DBP), and pulse pressure (PP) — have identified hundreds of loci associated with BP traits (5), but the causal genes remain largely unknown. Several genetic variants in the *PRDM6* locus on Chr5q23.2 have been associated with BP traits by genome-wide association studies (GWAS) in individuals of Western European (6), Finnish (7), and East and South Asian ancestry (8).

*PRDM6*-encoded protein is a histone modifier that is exclusively expressed in smooth muscle cells (SMCs) in adult life, reaching its highest levels in the SMCs of the aorta and other arteries (9–11) and is, therefore, a great candidate gene for arterial hypertension. During embryonic life, it is also expressed in the neural crest cells (NCCs) (12), a transient structure that provides inductive signals for organogenesis in diverse tissues, including renal stroma (13). Consequently, we embarked on examining the regulatory landscape of PRDM6, to characterize its function and its regulated network and to explore the relationships between the GWAS-sequence variations in this gene and BP traits. We used pairwise linkage disequilibrium (LD) measures to identify SNPs that are in LD with the GWAS sentinel SNPs in the *PRDM6* gene and used open-chromatin and histone-marks mapping to refine the locus. To identify the causal variants, we used CRISPR-Cas9–mediated gene editing and massively parallel reporter assay (MPRA) in HEK293T cells, which are epigenetically well-characterized human embryonic kidney cells that originate from NCCs (14) and abundantly express PRDM6. It is noteworthy that there is a strong overlap and functional conservation of *cis*-expression quantitative trait loci (*cis-*eQTLs) across different cell types (15–17). Our analysis led to the identification of *PRDM6* super enhancers that are in LD with lead SNPs in the third intron of *PRDM6* in a locus that is densely populated by binding sites for transcription factors (TFs), such as STAT1 and CEBPa/b. Interestingly, genetic variants in the *STAT1* gene itself have been associated with systolic hypertension (CVDKP; https://cvd.hugeamp.org).

We next focused on exploring the mechanisms by which PRDM6 regulates BP in vivo. We first examined SBP, DBP, and PP in mice with inducible disruption of the *Prdm6* gene in SMCs. Observing no difference in any of the BP traits between transgenic mice and WT littermates, we considered the developmental role of *Prdm6* in BP regulation. Strikingly, the kidneys of heterozygous SMC-specific knockout mice expressing Cre recombinase under the control of mouse smooth muscle cell protein 22-α promoter (*Prdm6^fl/+^ SM22-Cre*) exhibited a markedly higher number of renin-producing cells and developed hypertension when fed a high-salt diet. The exploration of the disease pathways by bulk RNA sequencing of the aorta led to the identification of a network of Prdm6-regulated genes that have been identified as GWAS loci for hypertension. Among these genes, *Sox6* was of particular interest since genetic variants in the *Sox6* locus have been associated with BP traits in diverse populations (18) and it has been reported to be a modulator of renin levels in the kidney (19). Further investigations showed that Prdm6 increases renin-producing cells in the embryonic kidney by upregulating Sox6. Taken together, these findings identify PRDM6 as a hub for a network of genes that regulate BP and characterize PRDM6 as a critical regulator for renin-dependent hypertension.

## Results

### Delineation of the regulatory landscape of prdm6 gene.

**Figure 1:**
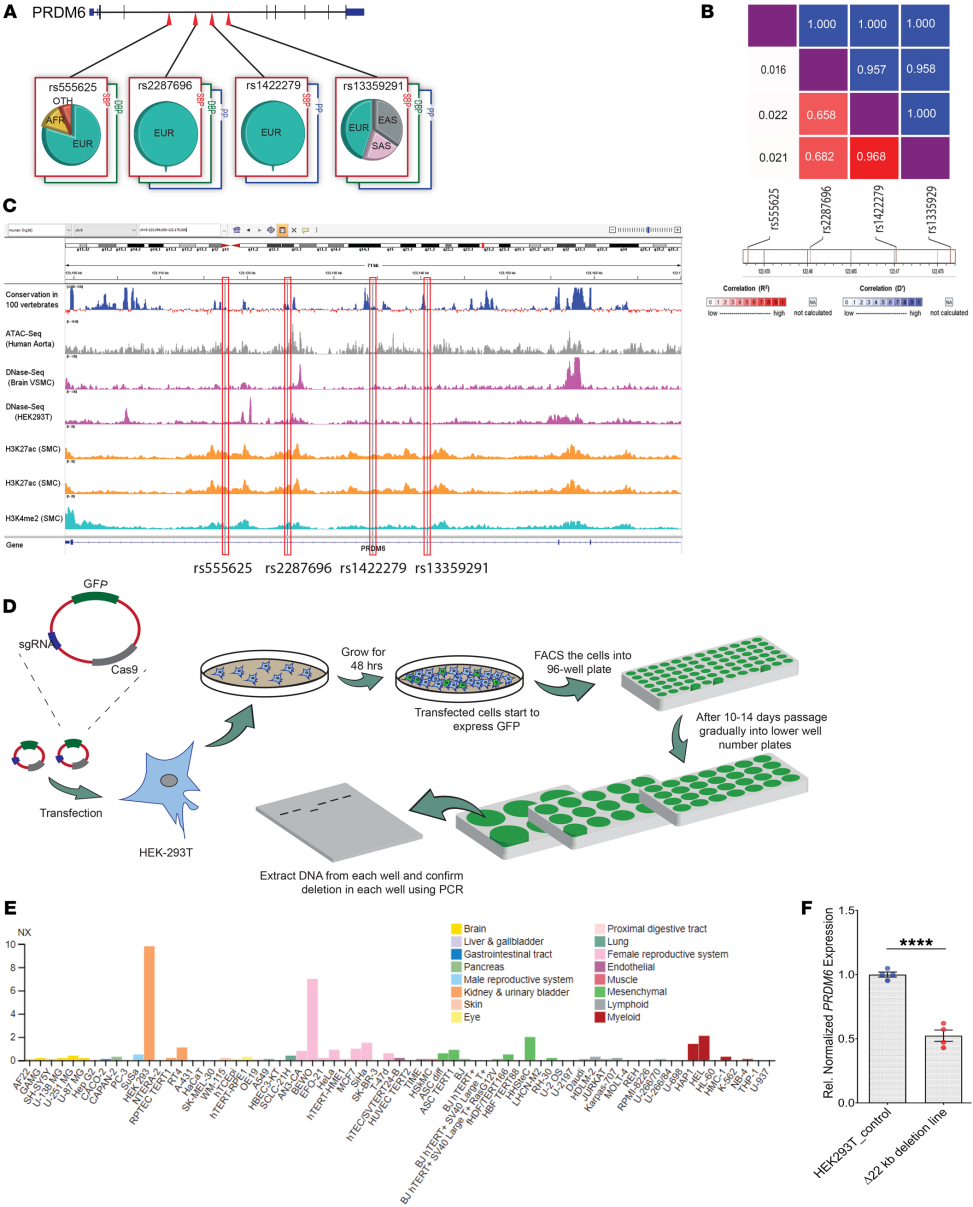
GWAS lead SNPs in PRDM6 intron 3 and their CRISPR deletion. (**A**) Genetic variants in PRDM6 gene discovered by GWAS of BP traits. (**B**) LD structure of rs13359291, rs2287696, rs1422279, and rs555625 in the East Asian population. R^2^ and D’ for each SNP pair is represented inside corresponding boxes. (**C**) Relationship between lead SNPs, open chromatin regions, and histone marks. (50, 51, 52, 53, 54). (**D**) Schematic of CRISPR-Cas9–mediated deletion of the approximately 22 kb LD region in intron 3, encompassing all lead SNPs, HEK293T, *n* = 4 technical replicates (**E**) Expression of PRDM6 in different human cell lines (www.proteinatlas.org/). (**F**) Significant reduction of *PRDM6* mRNA expression upon deletion of the LD region compared with the WT sequence assayed by RT-qPCR, identifying the intronic region as an enhancer locus for PRDM6. *n* = 4. Unpaired, 2 tailed, *t* test, *n* = 4 technical replicates. ****P < 0.0001. NES, normalized effect size.

Several genetic variants in the PRDM6 gene on Chr5q23.2 have been significantly associated with BP traits in individuals of Western European (6), Finnish (7), and East and South Asian ancestry (8) (Figure 1A). rs13359291 (SBP *P* < 2.27 × 10^–24^; DBP *P* < 2.18 × 10^–17^; PP *P* < 48 × 10^–17^ and rs2287696 (SBP *P <* 4.79 × 10^–24^; DBP *P* < 5.36 × 10^–18^; PP *P* < 1.42 × 10^–16^) are associated with all 3 BP traits, while rs1422279 (SBP *P* < 4.45 × 10^–26^; PP *P* < 1.57 × 10^–30^) and rs555625 (SBP *P* < 1.49 × 10^–12^; DBP *P* < 2.80 × 10^–12^) are associated with SBP and one of the other BP traits (T2SKP; https://t2d.hugeamp.org/#). rs13359291 has been identified as a lead SNP by multiple studies. It is an eQTL for *PRDM6* in tibial arteries (*P* < 1.2 × 10^–7^) and aorta (*P* < 5.7 × 10^–4^) (GTEx database) (Table 1), with net effect sizes of –0.16 and –0.11 respectively. rs2287696, rs1422279, and rs555625 are eQTLs in the aorta (Table 1). Using LDlink, we carried out pairwise LD analyses using the East Asian genome, since the lead SNPs show their strongest effects in this population. The analyses showed that rs13359291, rs2287696, and rs1422279 are in LD with each (R^2^ > 0.65 and D` > 0.95) and are located in the third intron of PRDM6 (Figure 1B), in open chromatin regions in the aorta and near histone marks associated with active enhancers (Figure 1C). The lead SNP rs555625 is also located in an open chromatin region of the aorta and is marked by H3K27ac in SMCs (Figure 1C).

To establish an association between the LD region and *PRDM6* expression we deleted the approximately 22 kb DNA segment that encompasses all lead SNPs, using CRISPR-Cas9 editing in HEK293T cells (Figure 1D), a cell line of neural crest origin (13) that abundantly expresses PRDM6 (Figure 1E). We screened the sgRNAs (Supplemental Table 13; supplemental material available online with this article; https://doi.org/10.1172/JCI160036DS1) based on an efficiency score of greater than 60 and self-complementary scores of less than 1 to increase the gRNA efficacy and eliminated all gRNAs that recognize genomic loci with greater than 20% sequence similarity to the target sequence to minimize the off-target effect. The off-target effect was further curtailed by characterizing multiple independent deletion lines for downstream analyses. The deletion resulted in a significant reduction in *PRDM6* mRNA expression compared with the WT sequence assayed by RT-qPCR, identifying the intronic region as an enhancer locus for PRDM6 (Figure 1F).

### MPRA identifies the causal SNPs for hypertension.

**Figure 2:**
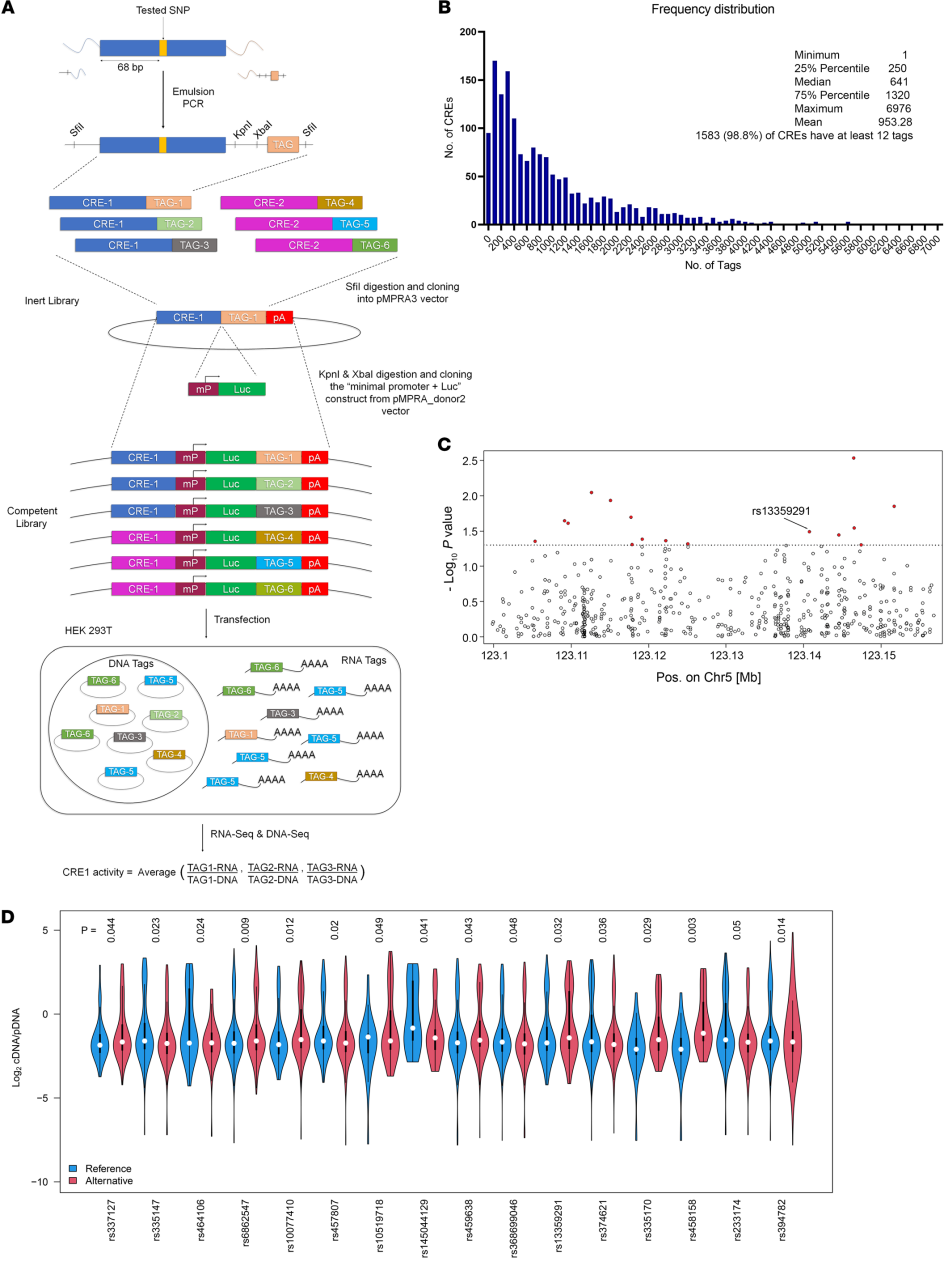
MPRA of PRDM6 intron 3 variants. (**A**) The schematic of MPRA of 336 common variants in the third intron of PRDM6 from generation of inert and competent libraries, cloning into MPRA vectors, transfection into HEK293T cells, and RNA & DNA sequencing and analysis. (**B**) Frequency distribution showing the number of tags associated with each MPRA fragment. Distribution statistics are represented. (**C**) Manhattan plot showing sixteen single SNPs that significantly altered the gene expression due to a single bp change with MPRA. (**D**) Violin plots of the 16 single SNPs showing allele-specific expression of the reporter gene. Statistics were carried out using 1-way ANOVA. *P* < 0.05 was considered significant.

We then carried out a MPRA and investigated the transcriptional regulatory potentials of 336 common variants in the third intron of PRDM6. Based on allelic permutations, we generated 1,602 unique MPRA fragments for our experiment. An oligo pool was synthesized based on the sequence information of these unique fragments, such that each fragment contained the genetic variant to be tested, flanked by 68 bp on each side. Additionally, common adaptors were incorporated into the oligos for PCR amplification and restriction enzyme–based cloning as described (20) (Figure 2A). Random barcode oligos were incorporated via emulsion PCR. The resulting inert library was sequenced (MiSeq paired-end 250 bp) to determine barcodes associated with each fragment. The library consisted of approximately 1.5 million barcodes distributed among 1,602 fragments (Figure 2B), such that greater than 98% of the fragments had at least 12 barcodes associated with differential activity analysis. The competent vector library was constructed by cloning a luciferase reporter gene and minimal promoter (minP) in between fragment and barcode and transfected into HEK293T cells. After 5 hours of incubation, cells were harvested and DNA and RNA were isolated and sequenced (HiSeq paired-end 150 bp).

**Figure 3:**
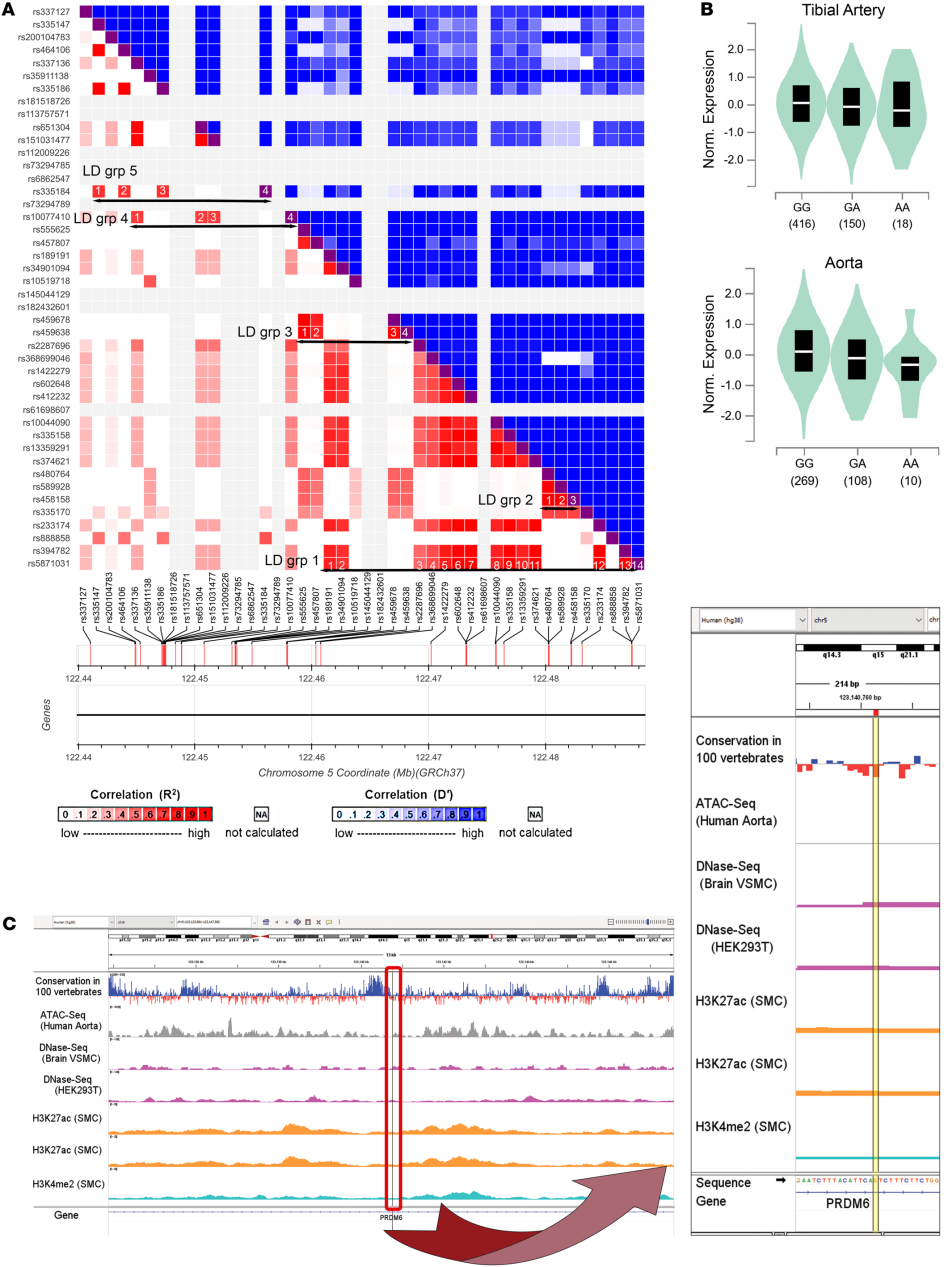
CRISPR deletion of SNPs with altered allele-specific expression and their location in different haplotypes. (**A**) 5 different LD groups (R^2^ > 0.70) in the East Asian population based on the haplotypes generated from lead GWAS SNPs and SNPs discovered by the MPRA. (**B**) Violin plots showing *PRDM6* expression in the tibial artery and aorta with different allelic combinations pertinent to rs13359291 (GTEx database, https://gtexportal.org/home/). (**C**) Location of the rs13359291 in relationship to open chromatin region of the aorta and histone markers and the sequence conservation in 100 vertebrates. The SNP region is zoomed in for clarity.

The assay revealed that 44 out of 336 SNPs in 37 reporter constructs, either alone or in a combinatorial fashion, conferred allele-specific regulation of *PRDM6* (Table 2 and Figure 2, C and D). Sixteen reporter constructs contained a single, 15 contained 2, and 6 contained 3 SNPs that showed allele-specific activity (Figure 2D and Supplemental Figure 1, A and B). To select candidate SNPs that are causal for BP we prioritized the SNPs that were lead SNPs or were in LD with the lead SNPs for BP traits. There were 5 different LD groups (R^2^ > 0.70) based on the haplotypes generated from lead GWAS SNPs and SNPs discovered by the MPRA (Figure 3A). Strikingly, the lead SNP rs13359291 itself showed significant allele-specific activity as a single SNP in the reporter construct (Table 2). This SNP is in an LD group (LD grp-1) with 13 other SNPs, including the lead SNPs rs1422279 and rs2287696 (Figure 3A). rs368699046 in the LD grp-1 also showed significant allele-specific activity when present as a single SNP in the reporter construct. rs10044090 and rs335158, present in this LD group, showed the strongest allele-specific activities in MPRA (Table 2). In LD grp-3 rs459638, rs459678 and rs457807, which were in strong LD with the lead SNP, rs555625 showed significant allele-specific activities (Figure 3A and Table 3). rs459638 independently exhibited significant allele specific activities but its effect size was greater when coexpressed with rs459678 and rs182432601 (Table 2).

We subsequently performed a colocalization analysis to determine whether the *PRDM6* variants (±750kb) showed shared effects on tissue-specific gene expression and traits related to blood pressure. Our colocalization analysis identified 4 signals of effects shared between *PRDM6* tissue-specific gene regulation and genetic associations with traits related to BP (Supplemental Tables 1–12). The variants identified were all in high LD (R^2^ > 0.9) with the *PRDM6* validated experimentally (Supplemental Figure 2). Specifically, rs1422279 showed multiple colocalization signals: (a) of *PRDM6* aorta–artery gene regulation with PP (LD proxy: rs1624823, PP_H4_ = 0.952) and SBP (LD proxy: rs1624822, PP_H4_ = 0.948); (b) between *PRDM6* tibial–artery gene regulation and PP (LD proxy: rs1624823, PP_H4_ = 0.921). Rs13359291 showed colocalization evidence between *PRDM6* tibial–artery gene regulation and SBP (LD proxy: rs10052206, PP_H4_ = 0.656).

**Figure 4:**
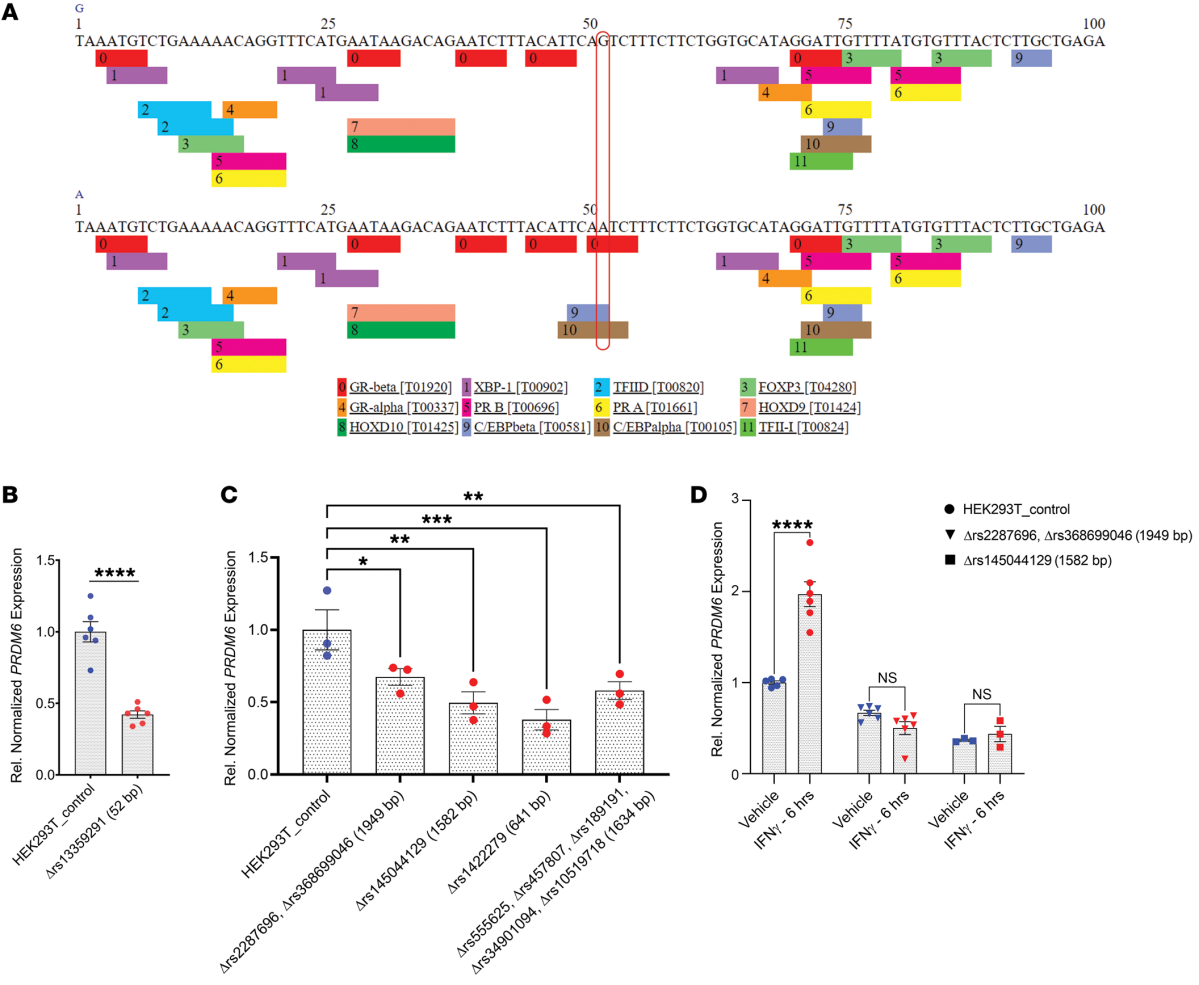
Screenings for transcription factor and enhancer identification. (**A**) Putative binding site for CEBP β and α based on PROMO silico motif finder. (**B**) Downregulation in *PRDM6* mRNA expression in the 52 bp CRISPR-Cas9 deletion encompassing rs13359291 compared with WT cells by RT-qPCR. Blue dots indicate control data points while red dots indicate experimental data points. (**C**) Downregulation in *PRDM6* mRNA expression in the depicted sized CRISPR-Cas9 deletions encompassing the mentioned SNPs, compared with WT cells by RT-qPCR. *n* > 3 independent replicates. ***P < 0.001, **P < 0.01, *P < 0.05. (**D**) IFNγ stimulation of HEK293T cells with and without CRISPR deletions encompassing STAT1 binding regions. Unpaired 2-tailed *t* test or 1-way ANOVA. ****P < 0.0001, ***P < 0.001. *n* > 3 independent replicates.

In summary, the MPRA identified, in total, 7 SNPs (Table 3) in LD grp-1 and LD grp-3 as functional SNPs. The finding suggested the existence of clusters of enhancers in the intron 3 of *PRDM6* gene, comprising an array of sequence elements that work together to regulate blood pressure. According to ChIP-Seq data from multiple tissue types available from ENCODE and Roadmap Projects (21) the super-enhancer locus spans a DNA sequence that is densely occupied by TFs, most notably STAT1 (Supplemental Figure 3). We subsequently confirmed the allele-specific effects of these SNPs in the endogenous genomic context by CRISPR-based genome editing in HEK293T cells, verified their deletion by Sanger sequencing, and assayed for *PRDM6* transcripts using RT-qPCR. We first deleted rs13359291, since it is associated with all BP traits, is a lead SNP in multiple GWASs, is an eQTL for *PRDM6* in the tibial artery and aorta (Figure 3B), and is located in an open chromatin region of the aorta (Figure 3C), albeit being only partially conserved (Figure 3C, zoomed). sgRNAs were designed to delete the 52 bp region encompassing rs13359291 and independent deletion lines were verified as previously described (Supplemental Table 13). PROMO, an in silico motif finder tool, predicts rs13359291 to be a putative binding site for CEBP β and α (Figure 4A and Table 4), which are known regulators of the renin-angiotensin-aldosterone system (RAAS) (22, 23). Using RT-qPCR we found a significant downregulation in *PRDM6* mRNA expression in the CRISPR-Cas9-deleted cells compared with WT cells (Figure 4B). We then deleted rs457807, which showed allele-specific activity in MPRA, and, together with the lead SNP rs555625, is in LD grp-3. Interestingly, we observed a significant change in *PRDM6* expression with the deletion of rs457807 as well (Figure 4C).

Taken together, these findings solidified the presence of a super-enhancer in intron 3. ChIP-Seq analysis according to ENCODE has identified a cluster of binding sites for STAT1 in this locus. Interestingly, a splice region variant in *STAT1* (rs2066804) has been associated with reduced BP (CVDKP. https://cvd.hugeamp.org). Consequently, we focused on SNPs that showed differential expression and are located within the STAT1 binding sites. We first deleted an approximately 2 kb STAT1 binding region in the LD grp-1 encompassing rs368699046 (Table 2) and in LD with the 2 lead SNPs rs13359291 and rs2287696 (Table 3). This deletion significantly reduced *PRDM6* expression in comparison with the WT control (Figure 4D). Next, we deleted rs145044129, which also showed allele-specific activity in MPRA and is flanked by a STAT1 binding region on the third intron of *PRDM6*. CRISPR-Cas9–mediated deletion of rs145044129 along with STAT1 binding locus reduced *PRDM6* expression significantly as well (Figure 3C). Since interferon-gamma (IFNγ) stimulates pSTAT1-dependent gene activation (24), we proceeded with IFNγ (100 ng/mL, 4 hours) stimulation of HEK293T cells with and without CRISPR deletions encompassing STAT1 binding regions. The results showed that the expression of *PRDM6* is increased upon stimulation with IFNγ in WT HEK293T cells, while no significant changes in both CRISPR-deleted lines were noted (Figure 4D), highlighting the role of STAT1 as a TF regulating PRDM6 transcription. Taken together, our data suggest the PRDM6 expression is regulated by a super-enhancer that is located in intron 3 and contains binding sites for multiple TFs, including STAT1.

### The heterozygous SMC-specific Prdm6-knockout mice develop hypertension in response to a high-salt diet.

**Figure 5:**
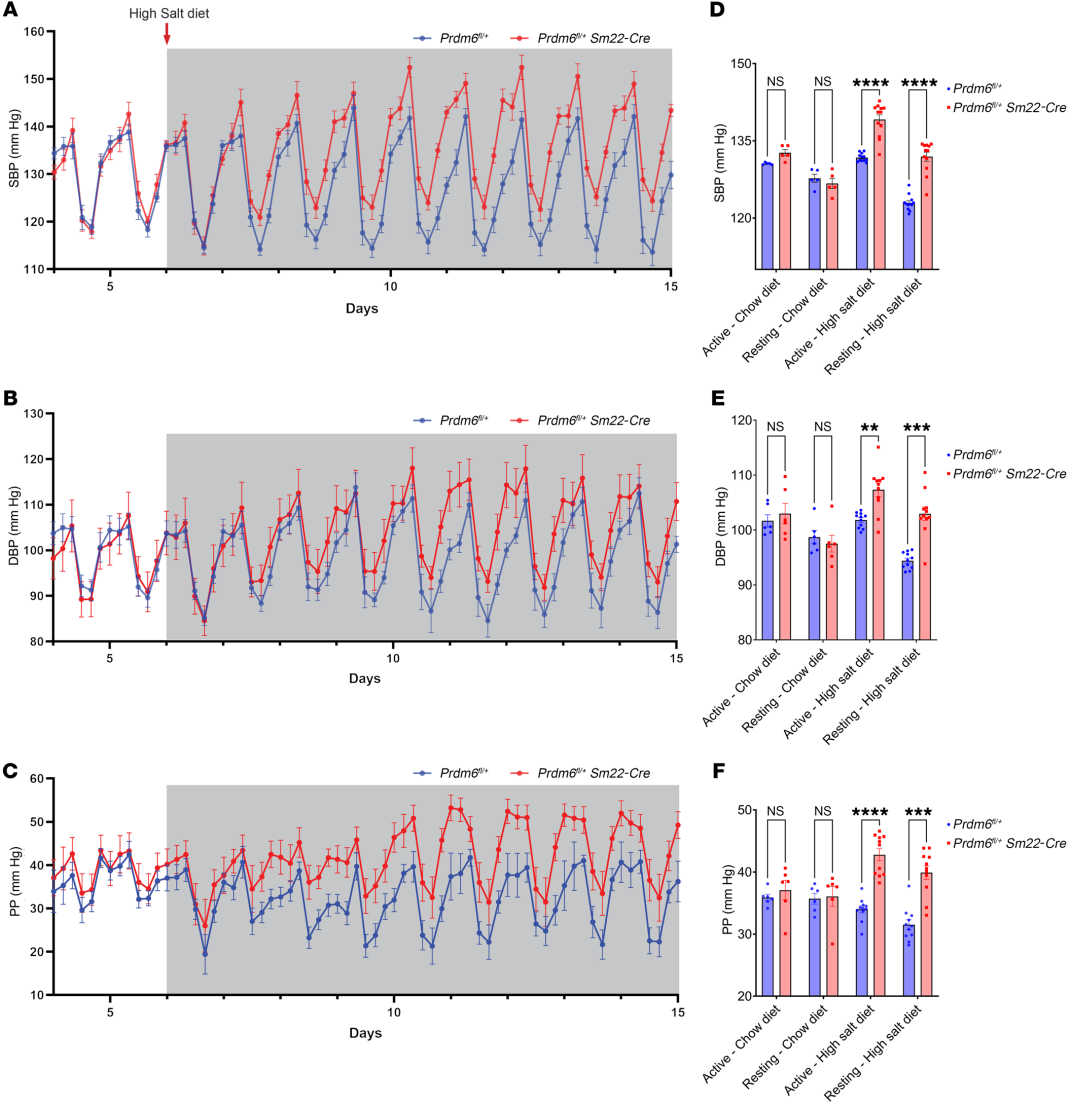
Invasive measurement of BP indices in *Prdm6^fl/+^ SM22-Cre* and WT mice. (**A**–**C**) SBP, DBP, and PP of *Prdm6^fl/+^ SM22-Cre* and *Prdm6^fl/+^* mice. Mice were on a chow diet (day 4–5) and a high-salt diet (day 6–14). (**D**–**F**) Changes of all 3 traits during the resting (daytime) and active (nighttime) phases under different dietary conditions. Multiple, paired, 2-tailed *t* test. ****adjusted *P* < 0.0001, ***adjusted *P* < 0.001. *n* > 5–11 mice per group.

We proceeded to examine PRDM6-regulated disease pathways in vivo by generating inducible conditional SMC *Prdm6–*knockout mice. We intercrossed *Prdm6^fl/fl^* female mice on C57BL/6J background with same-background SMMHC-CreER^T2^ male mice and expressed CreER^T2^ under the control of the mouse smooth muscle myosin heavy polypeptide 11(*Myh11*) promoter/enhancer regions on the Y chromosome. All mice were fed a chow diet and received tamoxifen (100μg) injections for 3 consecutive days (P1–P3). The 15- to 20-month-old mice underwent invasive telemetry-based BP monitoring and were introduced to a high-salt diet (8% NaCl; Research Diets) on the sixth day of BP monitoring. Surprisingly, no change in SBP or DBP was noted in transgenic mice compared with their WT littermates (Supplemental Figure 4A). We hypothesized that Prdm6 developmentally regulates BP and decided to examine BP in heterozygous SMC-specific *Prdm6*-knockout mice, since homozygous mice die on approximately P1–P2 (12). Thus, 15- to 20-week-old heterozygous (*Prdm6^fl/+^ SM22-Cre*) mice fed with a chow diet were introduced to a high-salt diet and underwent telemetry-based BP measurements. Both female and male, WT and transgenic mice showed a rise in SBP and DBP when placed on a high-salt diet, but heterozygous KO mice showed a much greater rise in SBP, DBP, and PP compared with WT littermates (Figure 5, A–C), most dramatically during the resting phase (Figure 5, D–F) when, paradoxically, the mutant mice were more active compared with their littermate controls (Supplemental Figure 4B).

### Prdm6 controls BP variation by restraining renin-producing cells.

**Figure 6:**
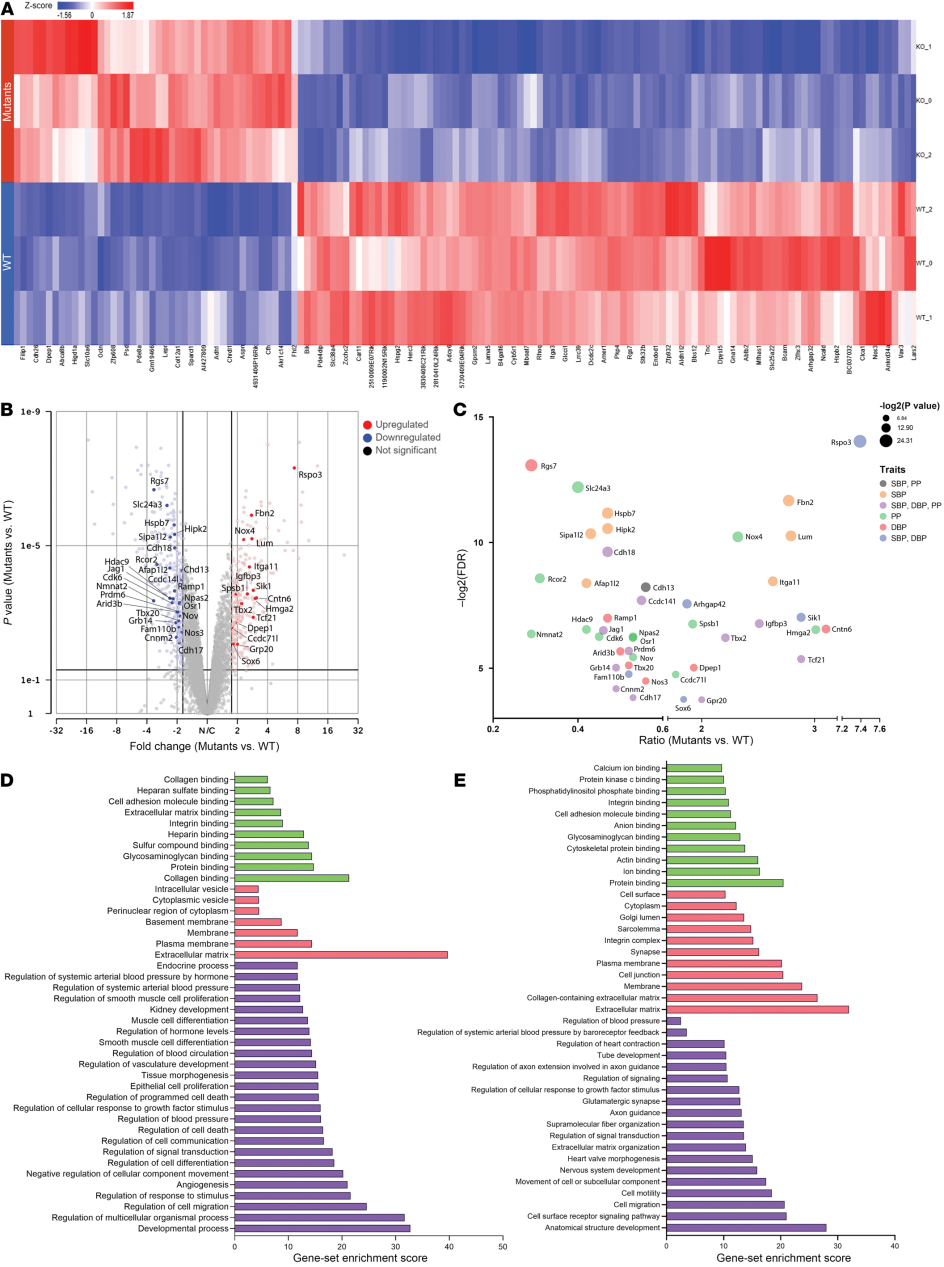
RNA-Seq analysis of *Prdm6^fl/fl^ SM22-Cre* mice aortic tissue. (**A**) RNA-Seq–hit map of homozygous SMC-specific knockout (*Prdm6^fl/fl^ SM22-Cre*) mice aortic tissue and their littermate controls at P0.5. A total of 349 of the genes were upregulated, while 769 genes were downregulated in *Prdm6^fl/fl^ SM22-Cre* versus WT mouse aortas. (**B**) Volcano plot demonstrating genes with significantly altered expression in *Prdm6^fl/fl^ SM22-Cre* mouse aortas versus WT mouse aortas; the plot shows fold change versus *P* value; a selected number of genes associated with hypertension are annotated for better visibility. (**C**) A bubble plot representing aforementioned genes associated with different BP traits. (**D** and **E**) GSEA show many blood pressure-related processes to be significantly enriched in both up- (**D**) and down-regulated (**E**) genes.

To investigate the molecular mechanisms by which Prdm6 controls BP we embarked on performing an RNA-Seq of the aortic tissues of homozygous SMC-specific knockout mice (*Prdm6^fl/fl^ SM22-Cre*) and their littermate controls for RNA-Seq at P0.5. The analysis showed 1,118 differentially expressed transcripts in *Prdm6^fl/fl^ SM22-Cre* compared with WT mice (*P* value < 0.05). While 349 transcripts were upregulated, 769 were downregulated in *Prdm6^fl/fl^ SM22-Cre* compared with WT mouse aortas (Figure 6A). Strikingly, 43 genes were localized at the peak GWAS loci for BP traits (Figure 6B). A significant number of these genes were associated with multiple BP traits (Figure 6C). The gene-set enrichment analysis using PartekFlow software identified many BP-related processes that were enriched with both up- and down-regulated genes (Figure 6, D and E). Similarly, the analysis identified a number of pathways related to BP regulation, most notably the RAAS (Supplemental Figure 5).

**Figure 7:**
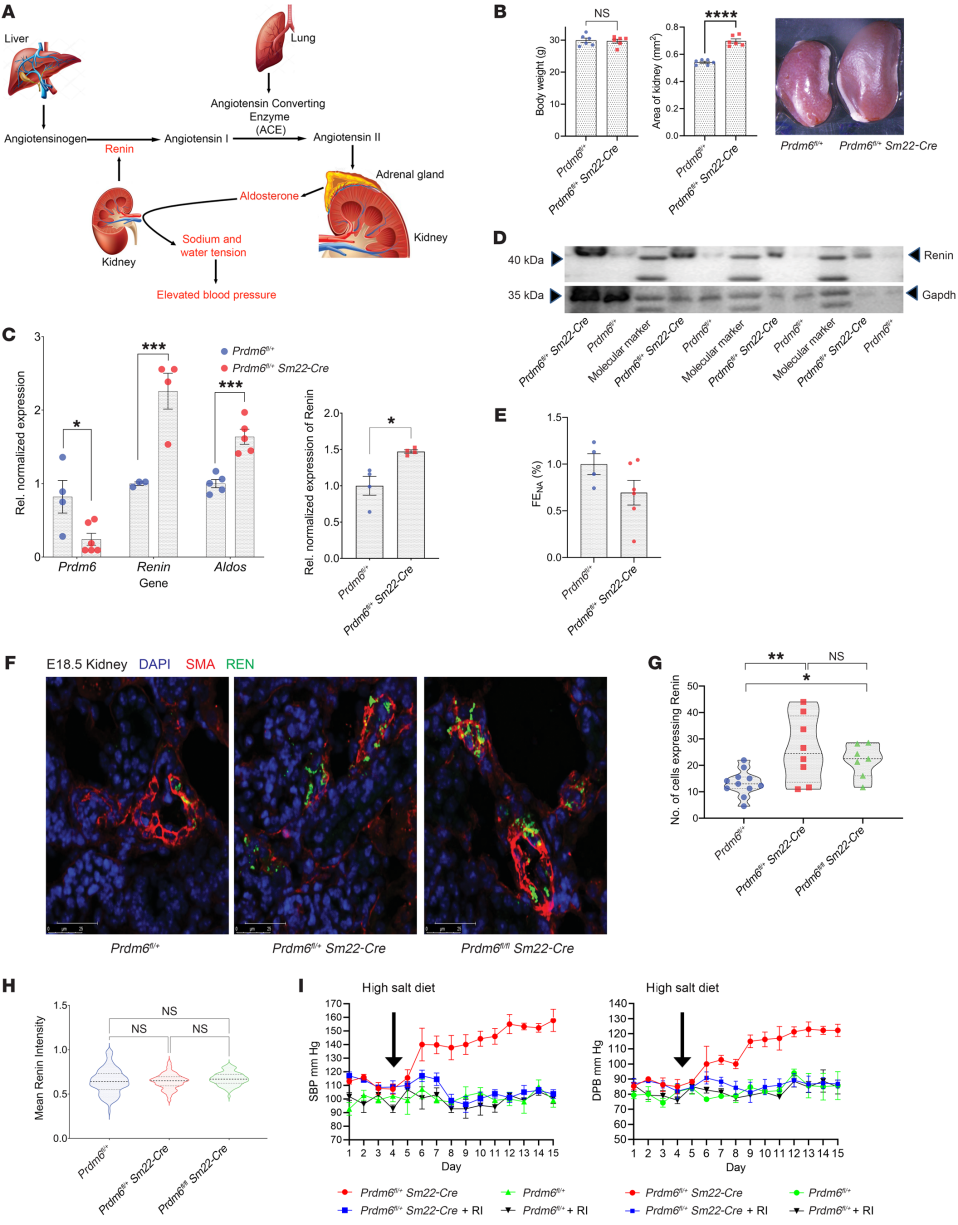
The increase in renin-producing cells and renin-aldosterone expression in *Prdm6^fl/+^ SM22-Cre* mice. (**A**) Schematic of renin-angiotensin-aldosterone–axis regulation. (**B**) Comparison of body weight and area of kidney between *Prdm6^fl/+^ SM22-Cre* and WT mice. *n* = 6 mice per group. (**C** and **D**) Higher renin mRNA and protein levels in the kidneys and aldosterone synthase mRNA levels in the adrenal glands of adult *Prdm6^fl/+^ SM22-Cre* mice versus WT littermates *n* = 4–6 mice per group. The quantification of the normalized Renin relative to GAPDH is shown in **C**. *n* = 4 mice per group. (**E**) The fraction of sodium excretion (FE_NA_) in *Prdm6^fl/+^ SM22-Cre* versus WT mice. *n* = 4–5 mice per group. (**F**) Renin protein levels in E18.5 kidney of *Prdm6^fl/+^ SM22-Cre* and *Prdm6^fl/fl^ SM22-Cre* mice and their corresponding WT littermates by immunofluorescence staining. (**G**) The number of renin-expressing cells per fixed area for all 3 genotypes. *n* = 7 mice per group (**H**) Mean renin intensity for the mentioned genotypes. (**I**) SBP and DBP measurements in *Prdm6^fl/+^ SM22-Cre* and WT mice on a high-salt diet with and without treatment with aliskiren. *n* > 6 mice per group. Data are shown as mean ± SEM. Kruskal-Wallis 1-way ANOVA. ****P < 0.0001, ***P < 0.001, **P < 0.01, *P < 0.05.

Renin is secreted from the juxtaglomerular (JG) cells of the kidney in response to hypotension, adrenergic stimulation, or low salt. These cells are precursors for arteriolar smooth muscle, mesangial cells, and interstitial pericytes (25) which are mainly within the walls of the afferent arterioles in macula densa (Figure 7A). *Prdm6^fl/+^ SM22-Cre* mutants have enlarged kidneys despite equal body weights (Figure 7B). Consistent with the RNA-Seq analyses results, we observed higher total renin mRNA and protein levels in the kidneys and also observed increased aldosterone synthase mRNA levels in the adrenal glands of adult *Prdm6^fl/+^ SM22-Cre* mice compared with their WT littermates (Figure 7, C and D). Accordingly, the fraction of sodium excretion showed a trend toward a reduction in *Prdm6^fl/+^ SM22-Cre* compared with WT mice fed with a high-salt diet (Figure 7E). These findings indicated increased RAAS activation in *Prdm6^fl/+^ SM22-Cre* mice compared with WT littermates. During embryogenesis, renin is expressed initially in the SMCs of larger intrarenal arteries, but in adult mice it is restricted to the terminal part of the preglomerular arterioles and coexpressed with α-smooth muscle actin (αSMA) (26). Strikingly, the immunofluorescence staining of *Prdm6^fl/+^ SM22-Cre* and *Prdm6^fl/fl^ SM22-Cre* mice kidneys for renin on E18.5 showed an increased number of renin-expressing cells compared with their corresponding WT littermates (Figure 7, F and G). Interestingly, we found no change in renin expression intensity per cell among the genotypes (Figure 7H), suggesting that Prdm6 does not control renin expression but regulates the number of renin-producing cells. Renin is normally suppressed in WT mice on a high-salt diet or with essential hypertension. To establish the causal role of renin in *Prdm6^fl/+^ SM22-Cre* mice hypertension we fed them a high-salt diet and simultaneously treated them with the renin inhibitor aliskiren by daily oral gavage (50 mg/kg). The BPs were measured for a total of 11 days on a high-salt diet by tail-cuff method. All mice had a normal growth curve (Supplemental Figure 6) and did not exhibit any apparent sign of distress throughout the experimental period, although it is plausible that the continuation of high-concentration salt could have resulted in adverse outcomes such as end-organ damage. As expected, the aliskiren was able to fully normalize the elevated BP in *Prdm6^fl/+^ SM22-Cre* mice but had no effect on the SBP or DBP of WT mice (Figure 7I). This finding supports the causal role of renin as a mediator for PRDM6 regulation of BP.

### Prdm6 controls renin-producing cells by transcriptional regulation of sox6.

**Figure 8:**
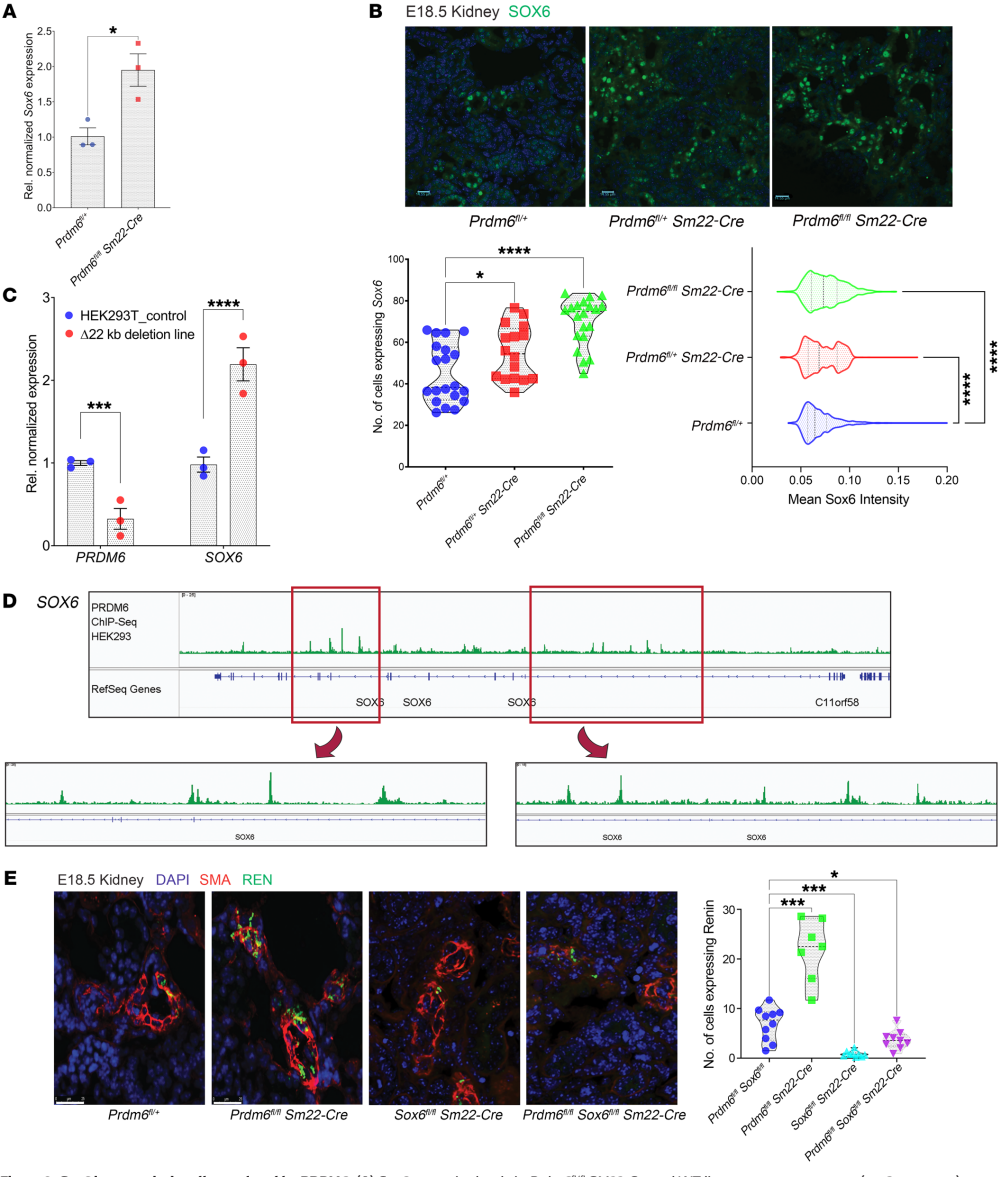
*Sox6* is transcriptionally regulated by PRDM6. (**A**) *Sox6* transcript levels in *Prdm6^fl/fl^ SM22-Cre* and WT littermate mouse aortas (*n* = 3 per group). (**B**) The immunofluorescence staining of Sox6 in heterozygous, homozygous *Prdm6*-knockout mice and their WT littermates. The number of cells expressing Sox6 (left) and mean Sox6 intensity (right) are shown underneath the figures (*n* = 3 mice per group and 20 fields per mouse). (**C**) *PRDM6* and *SOX6* expression level in HEK293T cells upon 22 kb CRISPR deletion of the *PRDM6* intronic region. Experiments were done in triplicates. (**D**) PRDM6 binding sites in *SOX6* gene in HEK293T cells according to ENCODE ChIP-Seq data (doi:10.17989/ENCSR892QHR). 2 binding loci are zoomed for clarity. (**E**) Immunofluorescent images of *Prdm6^fl/+^, Prdm6^fl/fl^ Sm22-Cre,* *Sox6^fl/fl^ Sm22-Cre* and *Prdm6^fl/fl^ Sox6^fl/fl^ Sm22-Cre* embryonic kidneys at E18.5 labeled with SMA (red) and renin (green) along with nuclear stain (blue). *n* = 3 mice per group and 10 fields per genotype. The plot on the right shows the quantification of a number of cells producing renin in 4 genotypes. Scale bars: 20 μm. IF figures: Kruskal-Wallis 1-way ANOVA, ****P < 0.0001, ***P < 0.001, *P < 0.05. RT-qPCRs, multiple unpaired, 2-tailed *t* tests. ****P < 0.0001, ***P < 0.001, *P < 0.05. Note: The *Prdm6^fl/+^* and *Prdm6^fl/fl^ Sm22-Cre* in Figure 7F are shown again in Figure 8E for comparison.

*Sox6* was one of the upregulated genes in RNA-Seq of *Prdm6^fl/fl^ SM22-Cre* mouse aortas (Figure 8A). Sox6 is highly expressed in JG cells, is upregulated by a high-salt diet, and triggers the recruitment and differentiation of renal mesenchymal stem cells to renin-producing cells (19). Strikingly, the *Sox6* gene locus has been associated with SBP, DBP, and PP (27). The immunofluorescent staining of heterozygous and homozygous *Prdm6*-knockout mice kidneys for *Sox6* showed increased numbers of Sox6-positive cells compared with the WT littermates (Figure 8B). Accordingly, a 22 kb CRISPR deletion of the *PRDM6* enhancer region resulted in a dramatic increase of the *Sox6* expression level in HEK293T cells (Figure 8C). Based on the ENCODE data set (doi:10.17989/ENCSR892QHR), PRDM6 binds to several regulatory regions of the *Sox6* gene in HEK293T cells (Figure 8D). Taken together, our findings indicate that Prdm6 regulation of renin-expressing cells is attained by transcriptional regulation of the *Sox6* gene. To support our finding, we disrupted *Sox6* in *Prdm6*-KO mice by intercrossing *Sox6^fl/fl^* and *Prdm6^fl/+^* and *Sm22-Cre* mice. While *Sox6^fl/fl^ Prdm6^fl/fl^ Sm22-Cre* were embryonically lethal, we were able to show a markedly reduced number of renin-producing cells in their kidneys compared with control (*Sox6^fl/fl^ Sm22-Cre*) mice on E18.5 (Figure 8E). Taken together, these findings indicated that Prdm6 developmentally regulated renin-producing cells by inhibiting *Sox6* expression.

### The mesodermal origin of the renin-producing cells.

**Figure 9:**
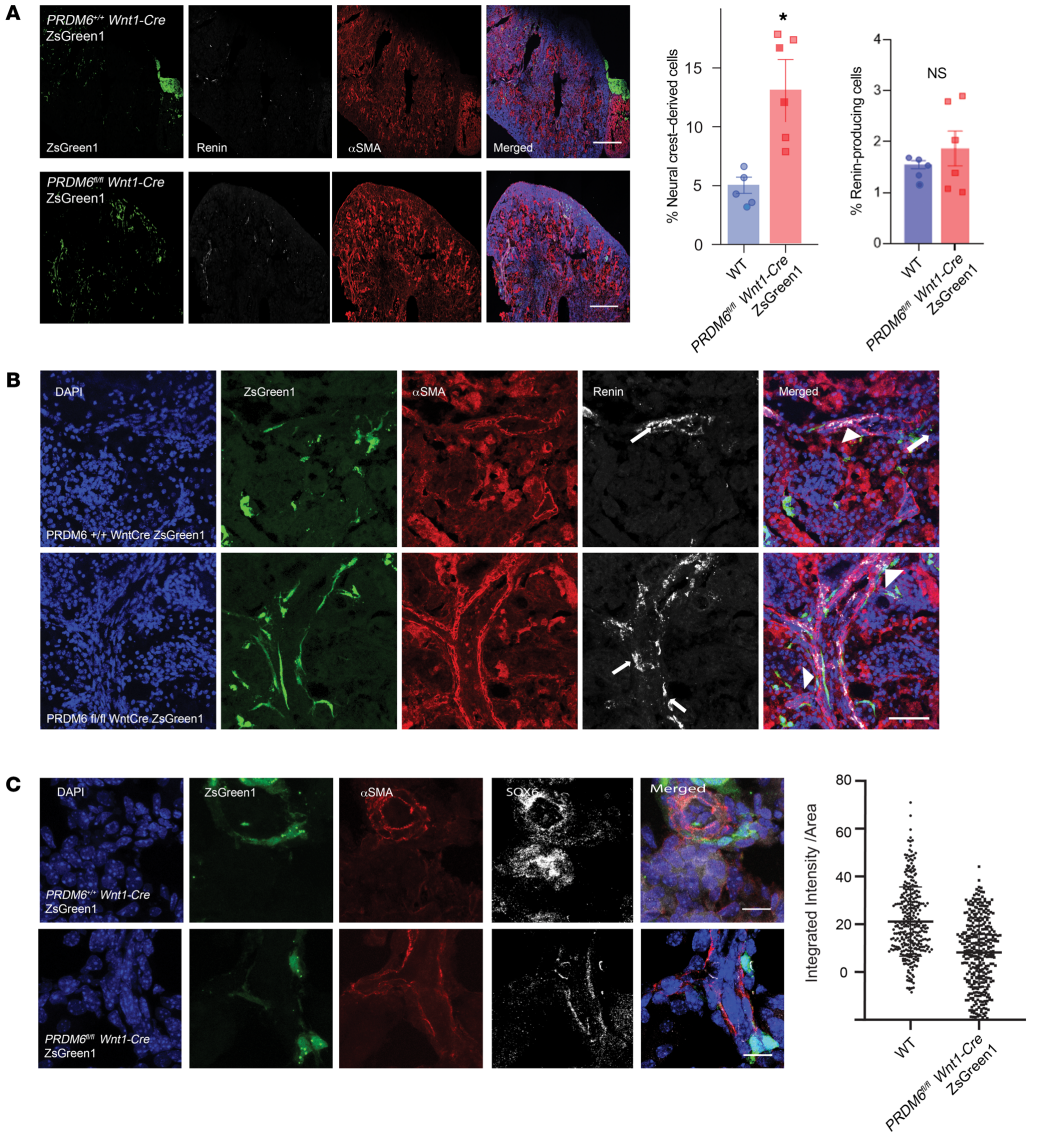
Fate mapping of neural crest PRDM6. (**A**) ZsGreen1 and renin-expressing cells in the kidneys of Prdm6^fl/fl^ Wnt1-Cre and WT littermates. The plot on the right shows the quantification of ZsGreen1 (NCC) and renin-positive cells. (**B**) Augmented part of panel **A** demonstrates the close proximity of ZsGreen1 and renin-expressing cells on the vascular wall of in *Prdm6^fl/fl^ Wnt1-Cre* kidneys marked by arrowheads. Arrows show renin. (**C**) The localization of Sox6 in relationship to αSMA and ZsGreen1positive cells (NCC) in *Prdm6^fl/fl^ Wnt1-Cre* kidneys and WT littermates. Scale bars: 20 μm. Unpaired 2-tailed *t* test; *P < 0.05. *n* = 6 mice per group in **A**–**C**.

**Figure 10:**
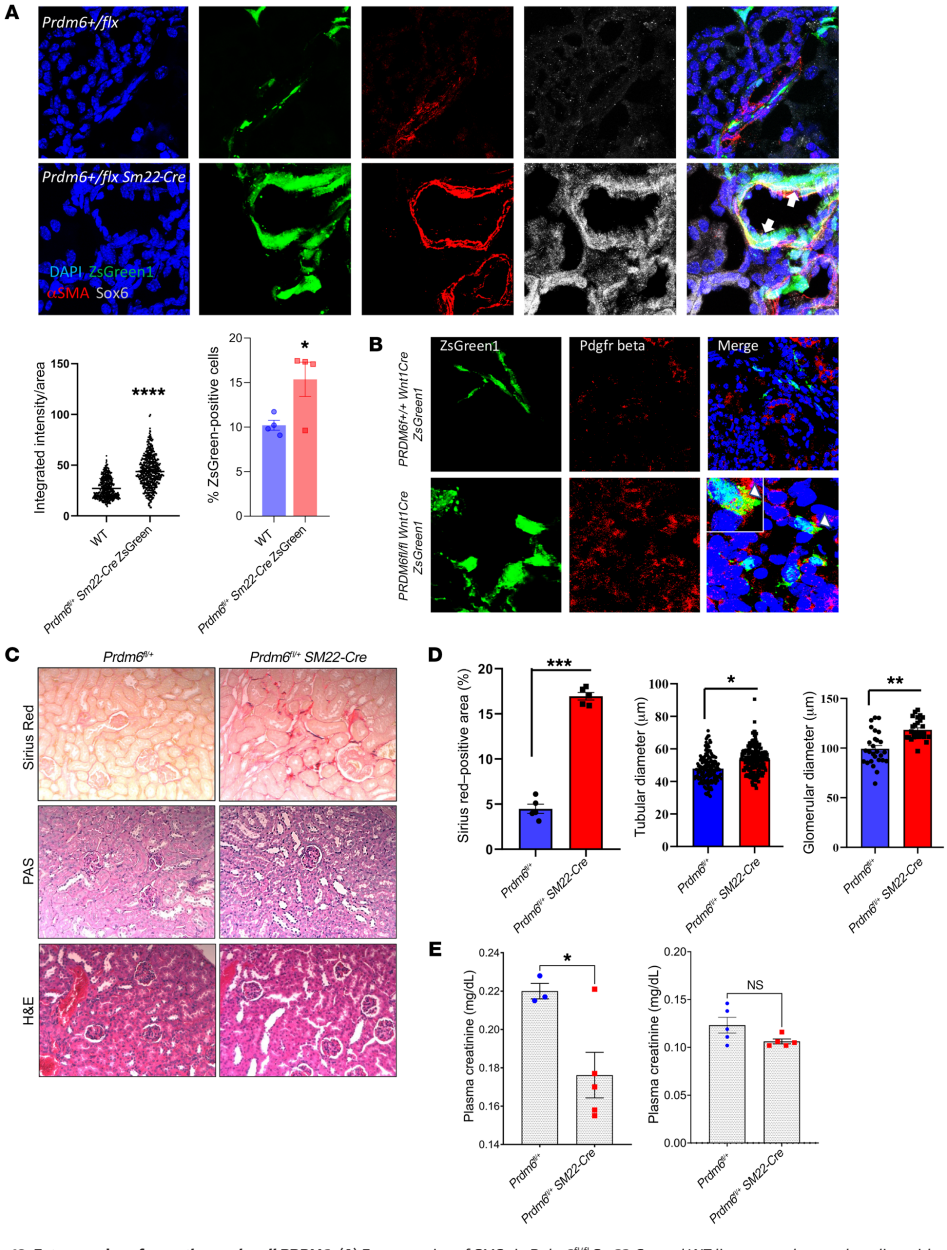
Fate mapping of smooth muscle cell PRDM6. (**A**) Fate mapping of SMCs in Prdm6^fl/fl^ Sm22-Cre and WT littermates by crossbreeding with ZsGreen1^fl/fl^ mice. (**A**) Colocalization of Sox6 and ZsGreen1 positive cells is shown; the integrated ZsGreen intensity per area and percent ZsGreen quantification of Sox6 in ZsGreen1 positive cells is compared between *Prdm6^fl/fl^ Sm22-Cre* and WT littermates. *n* = 500 cells per genotype. (**B**) Colocalization between PDGFRβ and ZsGreen1. *n* = 4 mice per genotype. (**C**) Sirius red, PAS, and H&E staining in 12 weeks adult heterozygous *Prdm6^fl/+^ SM22-Cre* mice kidneys compared with WT mice, *n* = 4 per group. (**D**) Tubular and glomerular diameters in *Prdm6^fl/fl^ SM22-Cre* mice versus WT littermates *n* = 6–100 measurements. Scale bars: 20 μm. (**E**) Creatinine levels in *Prdm6^fl/fl^ SM22-Cre* mice versus WT littermates on chow and high-salt diets, respectively unpaired 2-tailed *t* test; **** specifies *P* < 0.0001, **P < 0.01, *P < 0.05. *n* = 5 mice per group.

Sox genes play key roles in fate specification, cellular differentiation, and neural crest development during embryonic life (28). PR/SET(PRDM) family of proteins are also highly expressed in the NCCs (29), participate in transcriptional regulation via chromatin remodeling, are involved in temporal and spatial regulation of gene regulatory networks necessary for proper neural crest development in mice (30) and zebrafish (31), or are downstream targets of the TFs that regulate the formation of the neural crest (32). Prdm6 is expressed in cardiac NCCs and regulates SMC specification (12). Given that renin-secreting JG cells are of SMC lineage (26), we used fate mapping to examine if renin-producing SMCs in *Prdm6^fl/fl^ SM22-Cre* mice are derived from NCCs. To this end, we intercrossed *Prdm6^fl/fl^ Wnt1-Cre* and *ZsGreen1^fl/fl^* mice, which are both on C57BL/6J background and examined the presence of ZsGreen1 in the kidneys at E17.5. There were considerably greater numbers of ZsGreen1 positive cells in the kidneys of *Prdm6^fl/fl^ Wnt1Cre,* which localized mainly adjacent to the vessel walls (Figure 9A). The renin level was not significantly different and did not colocalize with ZsGreen1 in *Prdm6^fl/fl^ Wnt1Cre* or WT mice (Figure 9B). Surprisingly, there were reduced levels of Sox6 (Figure 9C) in *Prdm6^fl/fl^ Wnt1Cre* compared with WT mice. Only a small fraction of the Sox6 colocalized with ZsGreen1. To establish the role of mesodermal PRDM6 in renal Sox6 expression, we proceeded to fate map SMCs in *Prdm6^fl/fl^ Sm22-Cre* mice by crossbreeding them with *ZsGreen1^fl/fl^* mice. As shown earlier, we noted increased ZsGreen1 cells expressing Sox6 in *Prdm6^fl/+^ Sm22-Cre ZsGreen1* mice compared with *Prdm6+/+ Sm22-Cre ZsGreen1* (WT) mice (Figure 10A), indicating that loss of PRDM6 in SMCs increased Sox6-expressing SMCs of mesodermal lineage. Taken together, these findings show that loss of PRDM6 in the mesodermal — but not in NCC-derived cells — resulted in increased Sox6 levels in the kidney.

Because NCCs can differentiate into myofibroblasts (33), we examined whether the transformation of migrating NCCs into myofibroblasts and deposition of collagen contributes to the larger size kidneys in *Prdm6^fl/fl^ Wnt1-Cre* mice compared with WT littermates. There was modest colocalization of PDGFRβ and ZsGreen1-positive cells in *Prdm6^fl/fl^ Wnt1-Cre* mice, providing indirect evidence for their myofibroblast identity (Figure 10B). Accordingly, there was increased Sirius red staining as evidence for increased collagen deposition in the interstitium of 12 weeks *Prdm6^fl/fl^ SM22-Cre* (heterozygous) mice kidneys compared with WT mice (Figure 10C). Interestingly, STAT1, the upstream transcriptional regulator of PRDM6 expression has been shown to be protective against renal interstitial fibrosis after ischemia-reperfusion injury (34). Whether the interstitial collagen also contributed to the elevated BP, as shown in systemic sclerosis (35), could not be determined. In addition, enlarged glomeruli contributed to the larger kidneys and lower plasma creatinine levels in *Prdm6^fl/fl^ SM22-Cre* compared with WT mice (Figure 10, D and E). Improved GFR may reflect increased hydrostatic pressure due to the higher systemic blood pressure.

Taken together, we believe that our study establishes the role of PRDM6 as a hub for a network of genes that regulate BP in part by controlling the abundance of renin-producing cells.

## Discussion

In this study, we use a systems biology approach to establish a link between GWAS variants at the *PRDM6* gene locus, the altered expression of *PRDM6,* and the development of systolic and diastolic hypertension. By combining LD analysis, a high throughput reporter assay, and CRISPR-Cas9–mediated gene editing, we first mapped the regulatory landscape of the *PRDM6* gene and identified a super-enhancer locus that is a binding site for numerous TFs, most notably STAT1. Interestingly, genetic variants in the STAT1 locus and hypertension loci that are enriched for STAT1 binding sites have been associated with hypertension (36). We then demonstrated a link between reduced PRDM6 transcript levels and the development of hypertension in vivo. Strikingly, hypertension developed only when PRDM6 was disrupted embryonically, indicating the developmental role of PRDM6 in BP regulation.

The delineation of the disease pathways led to the discovery of PRDM6 as an embryonic regulator of renin-expressing cells and their differentiation into SMCs in the kidney. Renin is a component of RAAS that plays a central role in the regulation of BP and electrolyte homeostasis (37). In the developing kidney, the expression of renin is detectable at E*14.5* in a subset of SMCs in the arcuate arteries (26). Later, most renin-producing cells differentiate into arteriolar smooth muscle, mesangial cells, and interstitial pericytes, and, consequently, renin expression shifts from proximal to distal parts of the tree and remains restricted to a small segment in renal afferent arterioles known as JG cells (25, 38). The differentiated cells retain the memory to reexpress renin to maintain homeostasis under physiological (25) and pathological conditions (39) by mechanisms that are not fully understood. PRDM6 is a histone modifier that plays a critical role in VSMC specification (12). Our findings suggest that loss of PRDM6 impairs Sox6-dependent differentiation of renin-producing cells in mesodermal-derived SMCs, resulting in an increased number of renin-producing cells in the kidney. The potential role of PRDM6 in other tissues in the regulation of BP traits was not examined in our study.

The RNA-Seq analysis of the aorta suggested that PRDM6 functions as a multifaceted protein that regulates the expression of a number of genes located in GWAS loci for BP and thus, functions as a hub for a network of BP-regulating proteins. Among PRDM6-regulated gene networks, the *Sox6* gene was of particular interest, as its encoded protein regulates the recruitment and differentiation of renal MSCs to renin-producing cells (19). Accordingly, our transcriptomic analysis followed by in vivo genetic rescue studies established the causal role of Sox6 in mediating PRDM6-regulation of renin-producing cells.

Fate mapping studies have suggested the involvement of lumbosacral NCCs in kidney organogenesis (13) and their contribution to renal interstitial fibroblasts (33). Others have proposed that interstitial fibroblasts are derived from mesenchymal FoxD1-positive progenitor cells (40) or *NKD2*-positive pericytes and myofibroblasts (41). We have previously shown that PRDM6 is expressed in both mesodermal and NC cell-derived SMCs (12). By using fate-mapping we show here that loss of PRDM6 in Wnt1-positive NCCs does not alter renin expression but may result in a modest increase in PDGFR-positive NCC-derived cells and increased collagen deposition in the kidney. Whether renal interstitial collagen deposition contributes to increased BP (35) is not known. It is noteworthy that STAT1 has been shown to be protective against renal fibrosis during renal injury (34). In conclusion, we believe that our study establishes the role of PRDM6 as a master regulator of BP and as an attractive target for the treatment of hypertension.

## Methods

### Generation of SM22-Cre knockout mice.

The day of plug detection was considered E0.5. Prdm6-floxed mice were provided by Jürgen Ruland’s laboratory (Technical University of Munich, Munich, Germany) and were intercrossed to generate homozygous Prdm6-floxed mice (*Prdm6^fl/fl^*). Homozygous Prdm6-floxed mice (*Prdm6^fl/fl^*) were viable, fertile, and indistinguishable from control littermates. Homozygous mice *Prdm6^fl/fl^* were intercrossed with SM22-Cre transgenic mice expressing high levels of Cre recombinase in SMCs. While homozygous progenies *Prdm6^fl/fl^ SM22-Cre* died shortly after the birth from a patent ductus arteriosus *Prdm6^fl/+^ SM22-Cre* mice were perfectly healthy and developed high BP only after being placed on a high-salt diet. *Sox6^fl/fl^* transgenic mice were a gift from Monique Lefebvre at the Cleveland Clinic Lerner Research Institute (Cleveland, Ohio, USA).

### Invasive arterial pressure monitoring in mice.

Eight-week-old male Prdm6^fl/+^ SM22-Cr mice and the WT littermates were anesthetized and placed on a heating pad to maintain their temperature at 37.0–38.0°C. The tip of the radiotelemetric device TA11PA-C10 (Data Sciences Int.) was implanted into the common carotid artery toward the heart attached to a SI TA11PA-C10 transmitter placed in a pouch underneath the skin along the mouse’s flank. Mice were fed an 8% salt diet. The rationale for use of a high-salt diet was its higher success rate in causing hypertension in heterozygous loss of function mice in prior studies and the fact that other models such as Deoxycorticosterone acetate (DOCA) salt and angiotensin II (AngII) would have caused RAAS activation downstream from renin. The recordings of the heart rate and beat-by-beat arterial BP started 7 days after surgery for acclimation. Data were analyzed using the Dataquest ART version 2.1 (Data Sciences International).

### Tail cuff BP measurement.

Repeat BP measurements using the renin inhibitor aliskiren were determined in conscious *Prdm6^fl/+^ SM22-Cr* mice and the WT littermates using a noninvasive computerized automated tail-cuff system (Vistech BP-2000 BP Analysis System) after 15 minutes of daily training for 5 consecutive days. All mice were fed a high-salt diet and either received aliskiren oral gavage (50mg/kg) or water; an average of 3 10-cycles were used for BP calculation using BP-2000 software.

### Mouse embryo and tissue preparation and immunohistochemistry.

Mouse embryos were collected and incubated for 15–20 minutes in PBS containing 4% paraformaldehyde solution (Santa Cruz Biotechnology Inc.) at 4°C and after rinsing twice in ice-cold PBS incubated for 24 h in 30% sucrose/PBS at 4°C, followed by embedding in Tissue-Tek O.C.T. compound (VWR) in plastic molds on dry-ice until frozen and stored at -80°C. The tissue was sectioned into 5–10 μm slices in a cryostat and dried on Superfrost Plus slides (Thermo Fisher Scientific) for 12–16 hours at room temperature.

### Immunofluorescence.

5–10 μm frozen sections were stained using immunofluorescent antibodies. All primary and secondary antibodies were diluted at 1:300. In most cases only validated and previously published antibodies were used. Otherwise, no primaries, knockout mice, and tissues not expressing the proteins were used as controls. Fluorescence images were obtained by Zeiss 4 laser Confocal microscope or Leica sp8, and the intensities were measured using the same laser settings for each set of antibodies tested and quantified with Image J (NIH) and adjusted for the area. The following antibodies for immunostaining were used, anti-SOX6 antibody (Novus, Cat NBP1-85811), anti-SMA antibody (Abcam, Cat ab220179), and anti-renin antibody (Novus, Cat NBP1-31559).

Immunofluorescence image analyses for colocalization and intensity measurements were conducted using ImageJ (NIH) and CellProfiler (Broad Institute of MIT and Harvard) tools.

### Collagen staining.

5–10 μm fresh sections were stained using Vitroview Picro-Sirius Red Stain kit (VitroVivo Biotech) as described in the protocol. The sections are covered with Weigert’s Hematoxylin solution for 8 minutes, followed by Picro-Sirius Red stain for 60 minutes. Slides were washed with acidified water, water, ethanol (95% and 100%), and finally, xylene. Subsequently, slides were covered with a coverslip along with Permount. Slides were imaged under the Nikon 80i microscope system.

### Bulk RNA-Seq.

The RNA was isolated using Trizol, reverse-transcribed to cDNA, fragmented, and ligated with sequencing adaptors with 4 μL used as input for RNA library preparation. Sequencing libraries were generated using v2-Pico Input Mammalian kit(Takara) and quality controls were performed with the Fragment Analyzer high sense small fragment kit (Agilent Technologies, sizing range 50–1000 bp). Samples were pooled and loaded on the NextSeq 500 (Illumina) with a loading concentration of 1.2 pM at the Yale Center for Genomic Analysis (YCGA). The reads were trimmed for quality and length with a minimum base quality of Q30, and a minimum length of 45. The reads were aligned to the mouse UCSC reference genome mm10 (42, 43) using TopHat2 (44). The alignment data were converted to per-gene counts using cufflinks (45), and further analyzed using cuffdiff and R and visualized using R as described previously (http://www.r-project.org/index.html).

### CRISPR-Cas9–deletion line generation.

Vector construction, plasmid transformation, and clonal line recovery was carried out as described previously (46). In brief, sgRNA sequences designed using CHOPCHOP (https://chopchop.cbu.uib.no/) were cloned into the pSpCas9(BB)-2A-GFP vector as outlined. Then, plasmids were transfected into HEK293T cells, obtained from ATCC and allowed to recover. Percent-GFP expression was used to determine transfection efficacy. Clonal lines were recovered with serial dilutions as described in the protocol. The deletion lines were verified using PCR and sequencing. Multiple lines were characterized to determine consensus phenotype and proceeded with one of the lines.

### MPRA and data analysis.

We identified 336 common genetic variants located on the third intron (chr5:123,099,000–123,156,999, GRCh38) of the *PRDM6* gene. For each of these common variants, an oligo was designed such that the variant was in the center of a 137 bp oligo sequence. Some MPRA fragments contained additional genetic variants near the central variant. For such fragments, all possible allele combinations were planned to be tested. This study plan resulted in 1,602 unique MPRA fragments. Oligo pool generation, library construction, library transfection, sequencing, and data analyses were carried out as explained elsewhere (20). In brief, oligos were tagged with random, 16 bp barcode tags using emulsion PCR. This inert library was sequenced (Illumina MiSeq 2 × 250 bp) to establish oligo-barcode connections. A luciferase reporter gene and a minP were cloned in between oligo and tag, such that the barcode was incorporated into the 3’ end of the reporter gene. This competent library was then transfected into HEK293T cells using a Polyplus transfection kit and the 3’ end of the reporter gene was sequenced as plasmid DNA (pDNA), mRNA, and the corresponding cDNA libraries (Illumina HiSeq 2x 150 bp). Read counts were summarized by barcode tag, normalized by library size, and log_2_ transformed. As small data values showed a Poisson-like distribution, we determined a lower-end cutoff by plotting each library’s distribution and removing normalized values below 0.5. The cDNA values were normalized by their corresponding pDNA values to produce the activity of each barcode tag. Barcode tags were then ascribed to the fragment from which they originated, and fragments with fewer than 12 barcode tags were excluded from downstream analyses. We identified variants that affected MPRA activity by performing a student’s 2-tailed *t* test between the activity of barcode tags of any fragment that contained a variant, relative to those of the WT reference fragment.

### Western blot and quantification.

Western blot (WB) was performed using standard procedures and the membrane was developed by Supersignal West Pico (Thermo Fisher Scientific) and Supersignal West Femto (Thermo Fisher Scientific). The WB was quantified by ImageJ as described (47). The anti-renin antibody (Novus, NBP1-31559) and anti-GAPDH (Abcam, ab9485) were used.

### RNA extraction and quantitative reverse–transcriptase PCR.

RNA extraction and quantitative reverse–transcriptase PCR (RT-qPCR) were performed as described (48), with minor modifications. Tissues/cells were collected and frozen. Total RNA was isolated using RNeasy Plus Mini kit (QIAGEN) and cDNA was synthesized using Superscript II (Invitrogen). The RT product was amplified with SsoFast qPCR Supermix (Bio-Rad) in a Bio-Rad CFX96 Real-Time PCR System using pairs of gene-specific primers. The primers were as follows: Human-*ACTB*-Forward, CACTCTTCCAGCCTTCCTTC; Human-*ACTB*-Reverse, GATGTCCACGTCACACTTCA; Human-*PRDM6*-Forward, GGTGGGGAACCTAGTAAGTCG; Human-*PRDM6*-Reverse, ACCGTTGAAGGGACATTTAAGTT; Human-*SOX6*-Forward, CTGCCTCTGCACCCCATAATG; Human-*SOX6*-Reverse, TTGCTGAGATGACAGAACGCT; MOUSE-*Actb*-Forward, TGTGACGTTGACATCCGTAAAG; MOUSE-*Actb*-Reverse, GCAGTAATCTCCTTCTGCATCC; MOUSE-*Ren*-Forward, GTGGACATGACCAGGCTCAGTG; MOUSE-*Ren*-Reverse, CACCCAGAGGTTGGCTGAACC; MOUSE-*Aldos*-Forward, TGGCTGAAGATGATACAGATCCT; MOUSE-*Aldos*-Reverse, CACTGTGCCTGAAAATGGGC.

### NCCs fate mapping.

*Wnt1-Cre2* mice, stock number 022501(*B6.Cg-E2f1Tg(Wnt1-cre)2Sor/J*) and *ZsGreen1* (*Rosa-CAG-LSL-ZsGreen1-WPRE*, Jax 007906) were purchased from The Jackson Laboratory. For lineage tracing, *Prdm6^fl/fl^* mice were intercrossed with *ZsGreen1^fl/fl^* mice. To knock out *Prdm6* in *Wnt1*-positive NCCs, the *Prdm6^fl/+^ ZsGreen1^fl/fl^* mice were further intercrossed with *Prdm6^fl/+^ Wnt1-Cre2* mice.

### Mouse embryo and tissue preparation and IHC.

Mouse embryos and tissues were collected and rinsed in ice-cold DPBS and fixed by incubation for 15–20 minutes in PBS containing 4% PFA (Santa Cruz Biotechnology) at 4°C. They were rinsed twice in ice-cold PBS and incubated for 24 hours in 30% sucrose/PBS at 4°C, followed by Tissue-Tek O.C.T. compound (SAKURA) in plastic molds, and allowed to freeze on dry-ice and stored at –80°C. The tissue submerged in O.C.T. compound was sectioned into on 5μm–10 μm slices in a cryostat and dried on Superfrost Plus slides (Thermo Fisher Scientific) 12–16hrs at room temperature.

### Locomotor activity measurement.

Mice from 6–8 weeks old were habituated for 2 weeks and individually housed in cages with wheel-running activity continuously recorded for 2 weeks using the Clocklab6 tool (www.actimetrics.com). Locomotor activity was monitored at 5 minute binned intervals, and activity data were displayed as actograms, as described (49).

### Colocalization analysis.

Colocalization analysis to determine whether the *PRDM6* variants (±750 kb) showed shared effects on tissue-specific gene expression and traits related to BP. This analysis was performed with the *coloc* R package (PMID: 24830394) using aorta, coronary, and tibial artery tissues with gene expression data from GTEx Release V7 and GWAS summary statistics related to high BP (*n* = 458,554; PMID: 30940143), SBP, DBP, and PP from European-descent individuals (*n* = 757,601; PMID: 30224653). Evidence of colocalization between GWAS and eQTL signals was considered when the posterior probability of colocalization (PP_H4_) was less than 0.5. LD of the variants with evidence of colocalization with the *PRDM6* SNPs (i.e., rs13359291, rs1422279, rs555625, and rs2287696) prioritized in the previous experiments were calculated using LDlink (PMID: 26139635) and considering 1,000 Genomes Project European-descent populations as reference panel.

### Code and data availability.

All differential gene expression analyses from bulk and scRNA-Seq and their associated metrics have been deposited in NCBI’s Gene Expression Omnibus (GSE195590).

### Statistics.

In vivo studies included a minimum of 5 mice in each group. In vitro studies were carried out in more than 3 independent experiments. The comparison between different groups was done by a 2-tailed unpaired *t* test. The normalcy was tested by the Kolmogorov-Smirnov test. F-statistic was calculated to determine if variances were different between samples, and the *P* values were then Welch corrected. The comparisons between multiple groups were done by 1-way ANOVA. Mann-Whitney’s test was conducted for data that was not normally distributed. Fisher’s exact test was carried out for the continuous variables. The preparation of graphs and all statistical analyses, including 2-tailed Student’s *t* tests, and 2-way ANOVA (SigmaPlot). *P* < 0.05 was considered significant. Data are presented as mean ± SEM. Fluorescence images were evaluated using Image J software (National Institutes of Health).

### Study approval.

The present studies were reviewed and approved by the Institutional Animal Care & Use Committee (IACUC) at Yale. All studies in animals were conducted in accordance with the National Institutes of Health Guidelines for the Care and Use of Laboratory Animals.

## Author contributions

KLG conducted most experiments and acquired and analyzed data. LH conducted some of the experiments and acquired and analyzed data. TR bred mice and performed all of the revised experiments. SU analyzed MPRA data. JK assisted KLG with experiments. SM analyzed GWAS data. BCM performed the colocalization analysis. RP supervised the colocalization analysis. DRK assisted KLG with experiments. NG and CS assisted with the preparation of the MEPRA library and its quality control assessment. JPN supervised the analysis of MPRA data. AM designed and supervised the research studies and wrote the manuscript.

## Supplementary Material

Supplemental data

Supplemental tables 1-12

## Figures and Tables

**Table 4:**
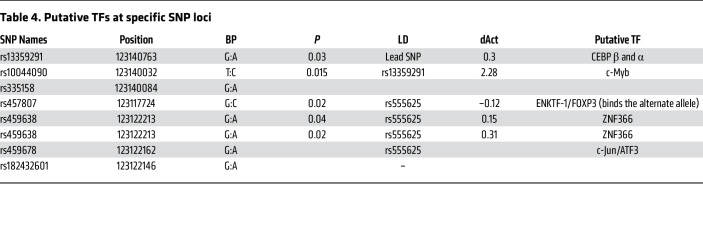
Putative TFs at specific SNP loci

**Table 3:**
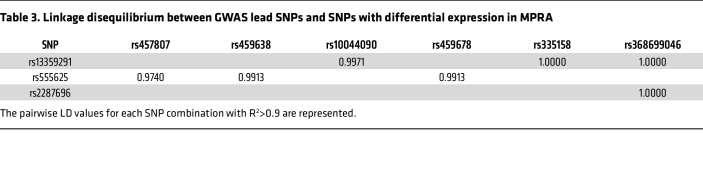
Linkage disequilibrium between GWAS lead SNPs and SNPs with differential expression in MPRA

**Table 2:**
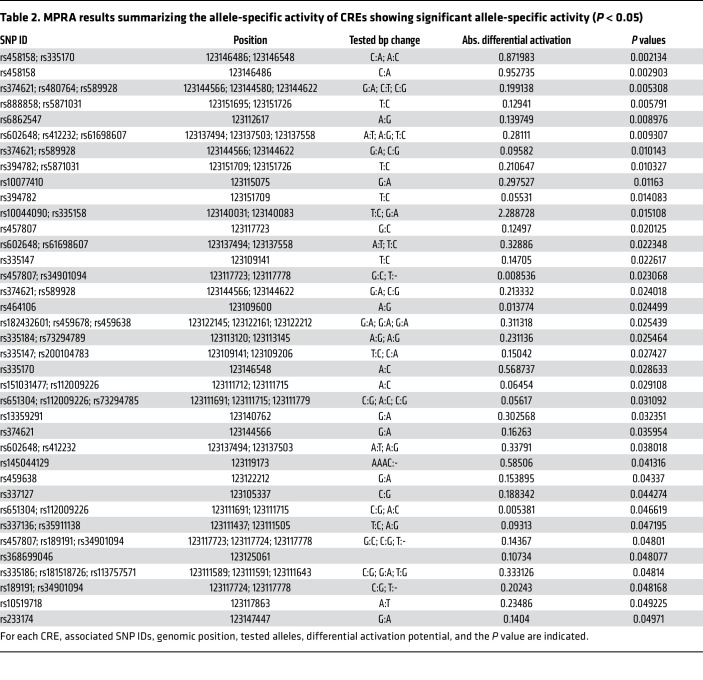
MPRA results summarizing the allele-specific activity of CREs showing significant allele-specific activity (*P* < 0.05)

**Table 1:**
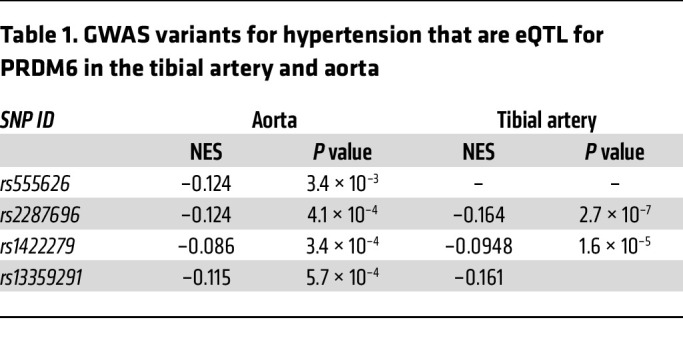
GWAS variants for hypertension that are eQTL for PRDM6 in the tibial artery and aorta

## Version 1 01/05/2023

In-Press Preview

## Version 2 02/15/2023

Electronic publication
