# Supplementary material for: A systems biology approach identifies the role of dysregulated PRDM6 in the development of hypertension
Source: J Clin Invest. 2023 Feb 15;133(4):e160036. doi: 10.1172/JCI160036 (PMC9927944; doi:10.1172/JCI160036)
Supplement: Supplemental data [file jci-133-160036-s273.pdf]

Table S13. Information on CRISPR-Cas9-based intronic deletions of PRDM6. Name of the deletions, two sgRNA sequences (PAM sequence is in **bold**) used to make the deletion, position & size of each deletion and SNPs that are being deleted (lead GWAS or significant CREs of MPRA) is indicated

Name	Upstream gRNA	Downstream gRNA	Deletion coordinates (hg38)	Size of deletion	SNPs deleted
Δ22kb	**CCG**TCCTGTGTTATGTCCAAACA	TGCTAGATGAGCTACAGATG**AGG**	chr5:123120267-123142393	22130bps	rs13359291<br>rs10044090<br>rs335158<br>rs602648<br>rs1422279<br>rs412232<br>rs368699046<br>rs459638<br>rs459678<br>rs145044129<br>rs61698607<br>rs182432601
Δrs13359291	AAAGTAAATGTCTGAAAAAC**AGG**	CAGTCTTTCTTCTGGTGCAT**AGG**	chr5:123140729-123140781	52 bps	rs13359291
Δrs1422279	**CCC**CCCATCTATTTAGAAAACAT	ATAGGCCGGAAACTGTGGTG**AGG**	chr5:123134514-123135155	641 bps	rs1422279
Δrs14044129	TGAGTTGTGTTCAAAAATGG**AGG**	TCTCACAGCTCACCATCCGG**AGG**	chr5:123119008-123120590	1582 bps	rs14044129<br>rs335163
Δrs2287696<br>Δrs368699046	**CCT**AAGTCCACTTTAGACACTCG	CTGAGGGGTAGTATCAATGA**GGG**	chr5:123124297-123126246	1949 bps	rs2287696<br>rs368699046
Δrs555625<br>Δrs457807<br>Δ18919<br>Δrs34901094<br>Δ10519718	TCGGATTGTCCTGCCCTAGA**AGG**	TGAGTTGTGTTCAAAAATGG**AGG**	chr5:123117374-123119008	1634 bps	rs555625<br>rs457807<br>rs189191<br>rs34901094<br>rs10519718

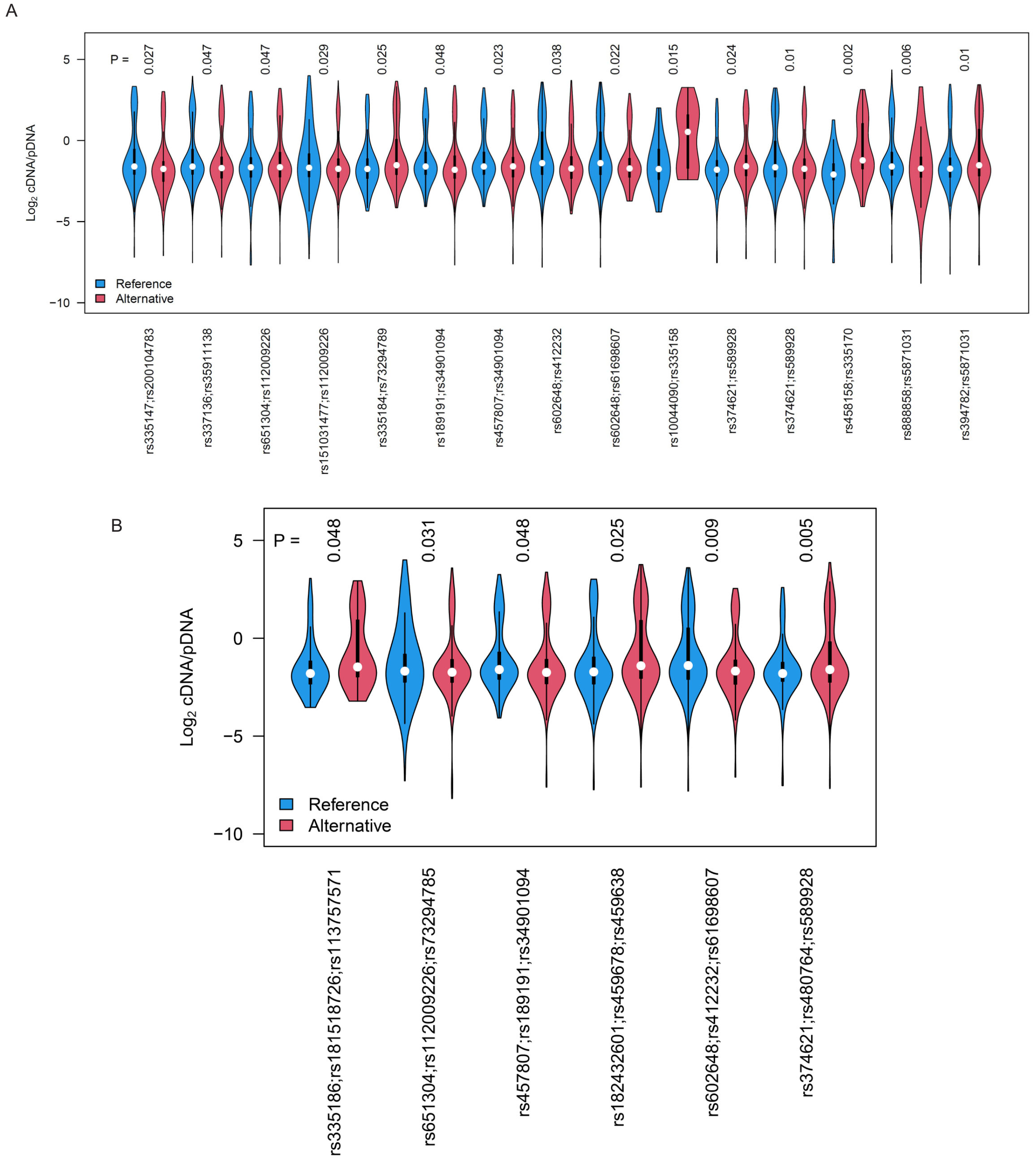

Figure S1. Massively Parallel Reporter Assay of PRDM6 intron 3 variants (cont.) Violin plots represent A) 15 CREs that showed allele-specific activation when two SNPs are simultaneously altered, while B) 6 CREs that showed allele-specific activation when three SNPs are simultaneously altered. An unpaired t-test was carried out and p-values are indicated in the figure.

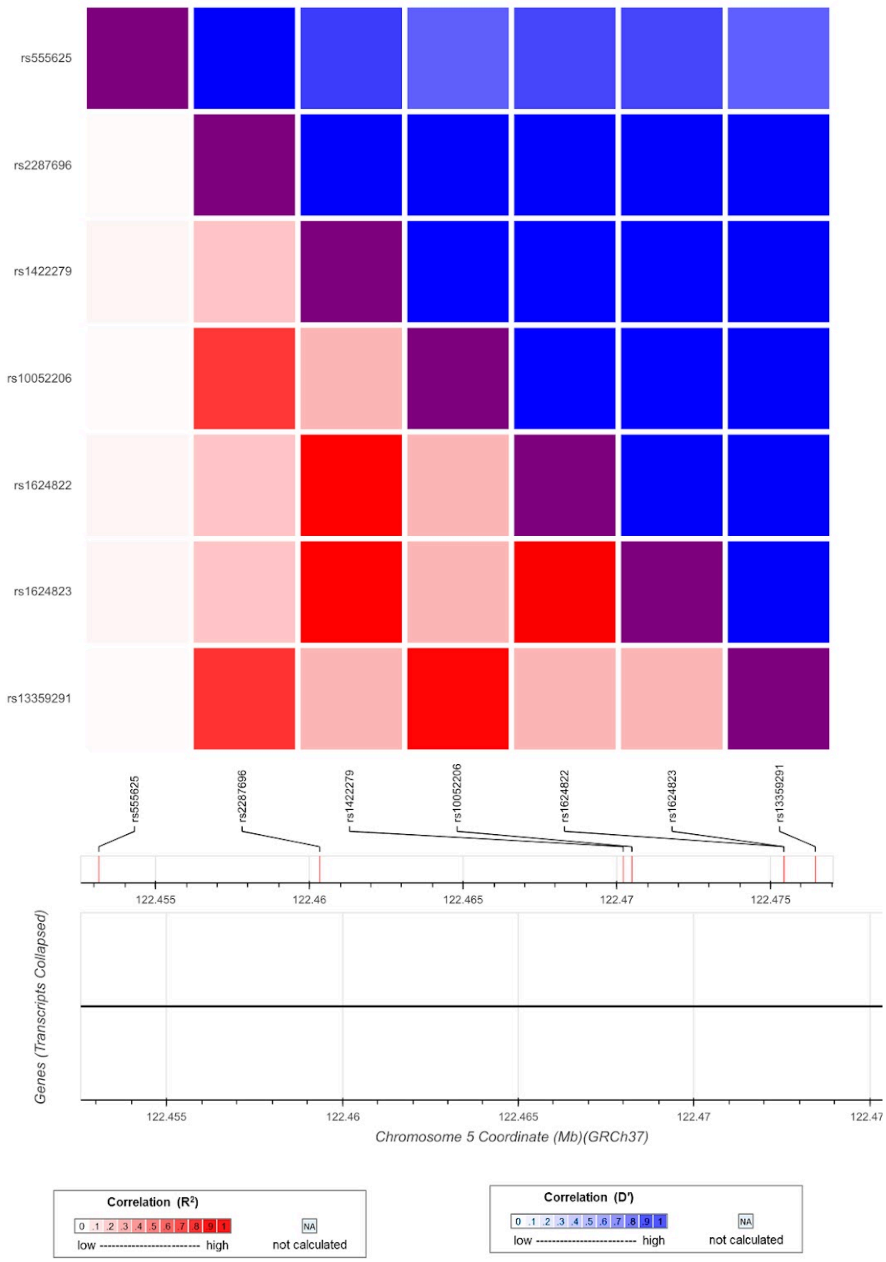

Supplementary Figure S2. Pairwise LD plot showing correlation (R2 and D prime) between individual SNPs showing strong evidence of colocalization across the *PRDM6* gene.

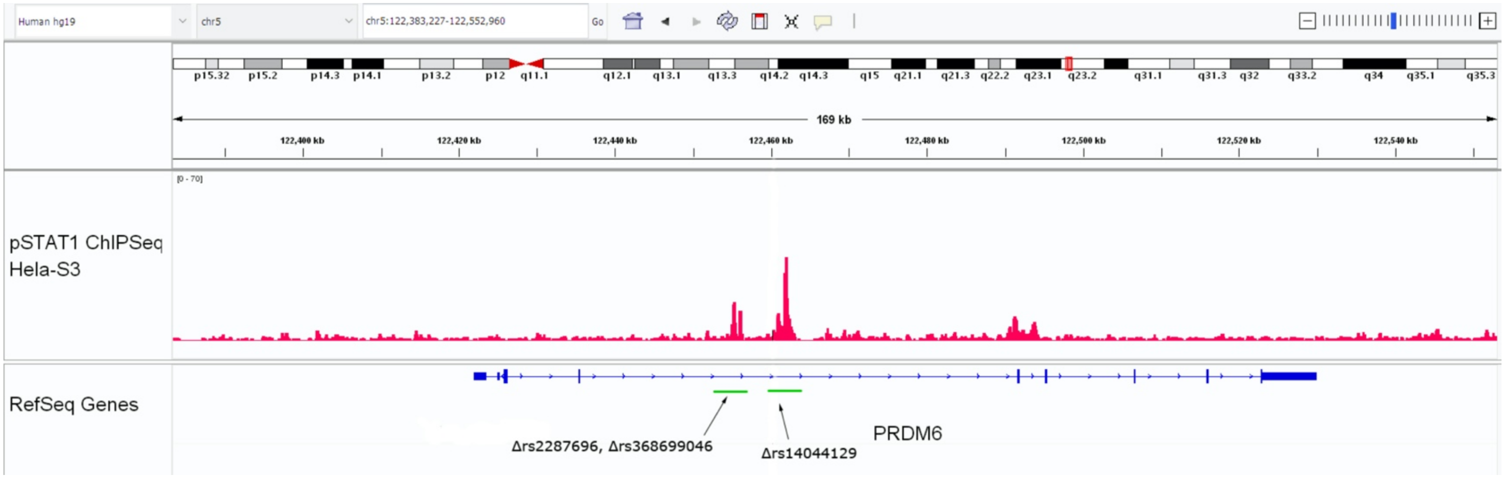

Figure S3. pSTAT1 occupancy on the intron 3 of PRDM6. pSTAT1 ChIP-Seq tract (doi:10.17989/ENCSR000EZK) showing that pSTAT1 bindings strongly on the 3rd intron of PRDM6. Two CRISPR deletions are indicated here for reference.

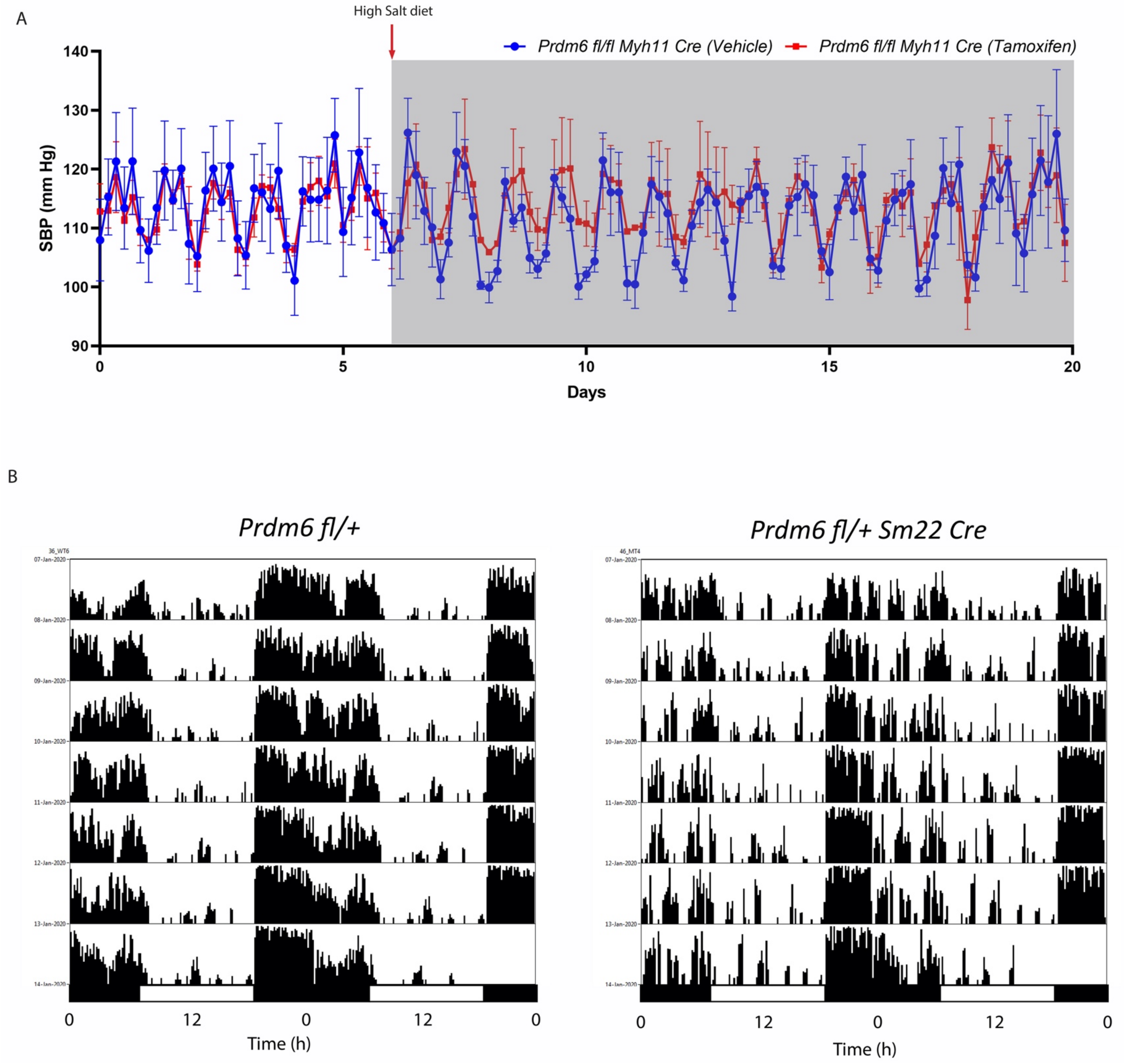

Figure S4. Mouse blood pressure and locomotor activity measurement experiments A) Invasive measurement of Systolic blood pressure (SBP) in *Prdm6fl/fl MyH11Cre* (Tamoxifen induced) and wildtype (corn oil) mice. Mice were introduced to a high-salt diet on the 5th day of the experiment. B) Locomotor activity of *Prdm6fl/+ Sm22Cre* and wildtype littermate mice were measured while they were exposed to 12:12hr light-dark cycles, while kept inside a light-tight box. BP experiments n=2 per each genotype and locomotor experiments n>8 per each genotype

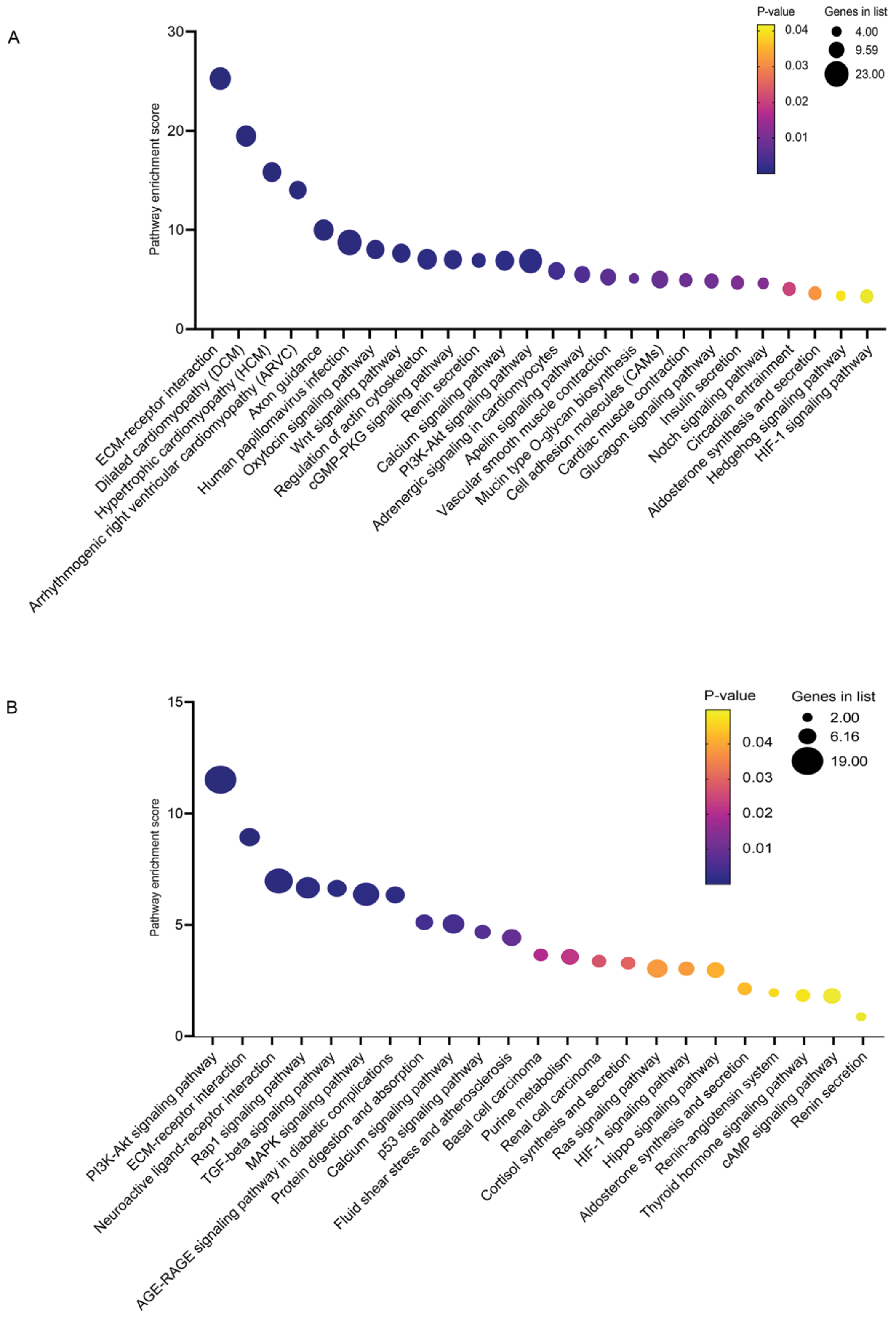

Figure S5. The enrichment of a number of pathways related to BP regulation in both up A) and down-regulated B) genes.

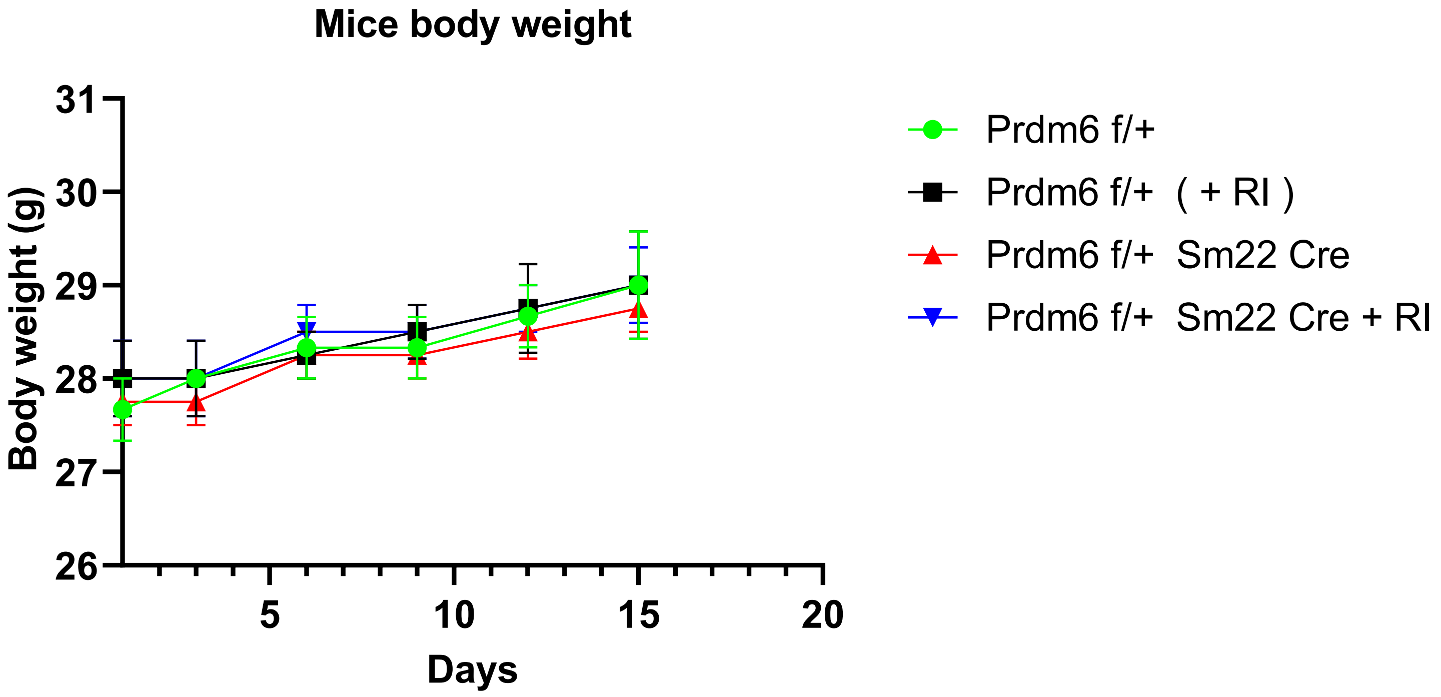

Figure S6. The body weight of Prdm6f/+ sm22Cre mice and the littermate controls on an 8% salt diet with and without treatment with aliskiren.
